# Accurate and efficient HiChIP interaction detection by modeling restriction enzyme cut site density as biological signal

**DOI:** 10.1093/bib/bbag292

**Published:** 2026-06-08

**Authors:** Weiyue Ding, Yang Zhou, Quanhong Liu, Yiyuan Guo, Chiping Zhang, Shuilin Jin

**Affiliations:** School of Mathematics, Harbin Institute of Technology, Harbin, Heilongjiang 150001, China; School of Mathematics, Harbin Institute of Technology, Harbin, Heilongjiang 150001, China; School of Mathematics, Harbin Institute of Technology, Harbin, Heilongjiang 150001, China; Department of Ophthalmology, First Affiliated Hospital of Harbin Medical University, Harbin, Heilongjiang 150001, China; School of Mathematics, Harbin Institute of Technology, Harbin, Heilongjiang 150001, China; School of Mathematics, Harbin Institute of Technology, Harbin, Heilongjiang 150001, China

**Keywords:** HiChIP, chromatin interactions, restriction enzyme, cut site density, chromatin accessibility modeling, statistical modeling

## Abstract

HiChIP enables high-resolution chromatin interaction mapping, but existing methods generally overlook restriction enzyme (RE) cut site density or treat it as a technical bias requiring normalization or removal, discarding chromatin accessibility information that distinguishes functional regulatory elements. Here we introduce sintHiChIP to address this methodological gap. sintHiChIP explicitly models RE cut site density as a biological signal and integrates Gaussian kernel smoothing with distance-dependent statistics, allowing detection of chromatin loops while capturing local regulatory heterogeneity. Moreover, the algorithm employs adaptive probability distributions to resolve inherent data overdispersion and sparsity dynamically. Validation against independent datasets and comparison with existing methods demonstrate that sintHiChIP reliably recovers canonical chromatin loops, exhibiting distinct superiority in regulatory H3K27ac environments and comparable accuracy in structural cohesin contexts. Notably, sintHiChIP achieves exceptional precision in predicting CRISPRi experiments and reveals highly coherent cell-type-specific genetic regulatory networks. Executing efficiently on standard workstations, our method delivers a promising analytical framework for functional 3D genomic studies.

## Introduction

Changes in chromatin folding can cause developmental diseases and cancer [1, 2]. Proximity ligation-based methods, such as Hi-C, provide the possibility of exploring the entire genome for chromatin contacts [3]. Although Hi-C generates genome-wide interaction profiles, deep sequencing is required to achieve high resolution and detect protein-associated contacts with higher efficiency [4, 5]. In contrast, ChIA-PET integrates chromatin immunoprecipitation (ChIP) with paired-end tag (PET) sequencing to detect long-range interactions between specific protein-bound regions in the genome, but it requires many cells [6]. HiChIP and PLAC-seq, methods based on IP and proximity ligation, reduce input cell requirements, and maintain target specificity [7, 8], enabling investigation of architectural proteins (e.g. CTCF, cohesin), histone marks (e.g. H3K27ac) in genomic architecture [9–11].

A key challenge in analyzing HiChIP data is addressing the non-uniform distribution of restriction enzyme (RE) sites. To date, computational methods have focused on removing these biases through various normalization and modeling strategies: hichipper adjusts ChIP-seq peak calling without significance testing [12]; MAPS (Model-based Analysis of PLAC-seq and HiChIP) employs zero-inflated models on contact matrices [13]; FitHiChIP adjusts for genomic distance and local coverage [14]; HiC-DC+ incorporates Hi-C normalization strategies to stabilize interaction profiles [15]; cLoops and cLoops2 use density-based clustering algorithms to detect contact clusters [16, 17]; MMCT-Loop combines peak- and cluster-based calling, filtering RE biases via permutation-based correction [18]. These approaches uniformly treat RE cut site density as a technical artifact requiring aggressive removal. This statistical penalty forces a severe biological cost. By normalizing away RE density heterogeneity, these methods actively discard potentially informative chromatin accessibility information.

Recent evidence confirms that RE site distribution correlates with chromatin accessibility, with higher cutting efficiency in open euchromatic regions such as promoters and enhancers compared to compact heterochromatin [19, 20]. Chandradoss et al. found that differential visibility in proximity ligation datasets fundamentally reflects regulated chromatin condensation states dynamically [19]. Consequently, standard normalization protocols eliminate the exact signals required to capture local regulatory landscapes.

Hence, we developed sintHiChIP to resolve this methodological conflict. sintHiChIP explicitly redefines RE cut site density as a biological signal rather than a technical bias, jointly integrating RE accessibility with distance, building on established probability models [21]. The framework dynamically accommodates local dispersion via adaptive binomial or negative binomial distributions, utilizing a Poisson fallback to mitigate extreme data sparsity. This statistical flexibility enables sintHiChIP to recover canonical peak-to-peak (P2P) loops and rescue peak-to-non-peak (P2N) interactions that existing approaches discard as noise. Benchmarking across GM12878 and K562 cell lines using Hi-C recovery, ChIA-PET, aggregate peak analysis, CRISPRi, and expression quantitative trait loci (eQTL) networks confirms sintHiChIP achieves superior performance in regulatory H3K27ac contexts and comparable results in structural cohesin HiChIP. sintHiChIP scales linearly with dataset complexity, processing over 200 million PETs in under five minutes while maintaining balanced computational efficiency.

## Materials and methods

### Dataset overview and experimental design

We benchmarked sintHiChIP using K562 and GM12878 H3K27ac HiChIP (regulatory context) [7] and GM12878 cohesin HiChIP (structural context) [22] against seven methods: statistical methods (FitHiChIP, HiC-DC+, MAPS, hichipper), a mix model (MMCT-Loop), and clustering methods (cLoops, cLoops2) (Supplementary Table S1). Performance was evaluated through: Hi-C and ChIA-PET loop recovery, aggregate peak analysis, CRISPRi-FlowFISH validation, eQTL pathway enrichment, and computational efficiency.

### Interaction modeling through RE cut site density

**Figure 1:**
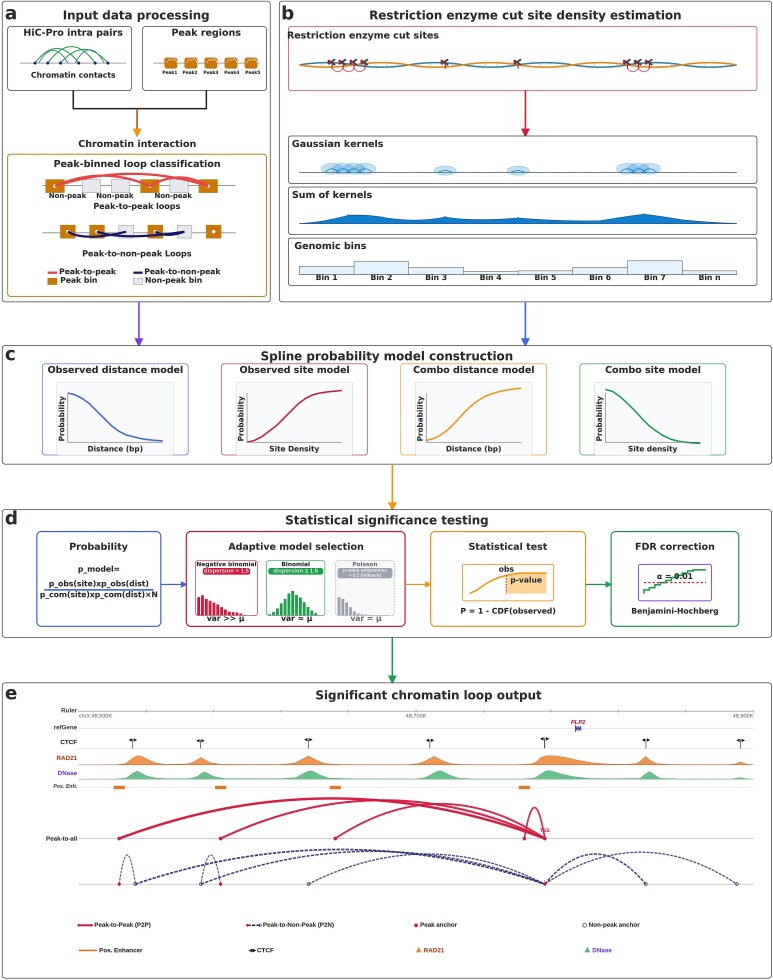
sintHiChIP workflow and statistical framework. (a) Input processing combines HiC-Pro valid pairs, peak regions, and RE cut sites. Genome-wide binning enables comprehensive detection of chromatin interactions where at least one anchor overlaps a peak region. (b) RE cut site density estimation using Gaussian kernel smoothing. (c) Four probability models establish empirical patterns and background expectations. (d) Adaptive distribution selection (negative binomial, binomial, or Poisson) with FDR correction. (e) Predicted loops at the PLP2 locus (chrX:48.5–49 Mb), including peak-to-peak (solid arcs) and peak-to-non-peak (dashed arcs) interactions, with CTCF binding sites, RAD21, and DNase tracks shown for context.

sintHiChIP identifies significant loops by integrating distance decay with RE cut site density. Starting from HiC-Pro [23] processed contacts and peak regions (Fig. 1a), the pipeline employs Gaussian kernel smoothing to generate continuous accessibility profiles (Fig. 1b). These signals inform a spline-based statistical engine (Fig. 1c) that adaptively selects Poisson, binomial, or negative binomial models based on variance-to-mean ratios and data sparsity (Fig. 1d). Final significance is determined via false discovery rate (FDR) correction [24] (Fig. 1e). To facilitate non-mathematical readers, a structured overview of the sintHiChIP pipeline is provided in Box 1 (Supplementary Methods).

GM12878 H3K27ac HiChIP analysis showed near-zero correlation between distance and RE cut site density (R^2^ = 0.002, *P* < 2.2 × 10^−16^, Variance Inflation Factor = 1.002; Supplementary Fig. S1a-b). Empirical HiChIP contact patterns fit robustly with the four probability distributions (Supplementary Fig. S1c-f), validating accurate capture of observed interaction frequencies across genomic scales by model predictions.

Stratified enrichment analysis across RE density deciles validated biological signal (Supplementary Figs S2-S3). H3K27ac HiChIP showed 2.40 times median site density gain (range: 0.05–24.1), while cohesin exhibited 1.24 times gain (range: 0.26–2.68). These context-dependent patterns (*P* < 2.2 × 10^−16^) confirm RE density encodes chromatin accessibility distinguishing regulatory from structural contexts.

### Anchor definition and interaction calling strategies

sintHiChIP divides the genome into a series of non-overlapping bins (5 kb by default) and models the standard P2P interactions (between bins containing peaks) along with P2N interactions (between peak anchors and non-peak anchors) to detect more meaningful signals.

### RE cut site density estimation

To better illustrate the distribution of RE cut site in the genome, we applied Gaussian kernel density estimation methods to estimate local density around RE sites. The local distribution of each RE site is modeled as follows:


(1) \begin{equation*} \rho =\sum_i\frac{1}{\sigma \sqrt{2\pi }}\exp \left(-\frac{{\left(x-{c}_i\right)}^2}{2{\sigma}^2}\right)\kern0.5em \end{equation*}

where ${c}_i$ and $\sigma$ represent the position of the RE site and influence range, respectively.

Total density at position:


(2) \begin{equation*} \rho (x)=\sum_i{\rho}_i(x) \end{equation*}

We separate the chromosome into fixed bins and calculate average densities:


(3) \begin{equation*} {\overline{\rho}}_b=\frac{1}{w}{\int}_{\left(b-1\right)w}^{bw}\rho (x) dx \end{equation*}

where $w$ represents the width of fixed bins and ${\overline{\rho}}_b$ calculates mean density in bin $b$.

### Statistical framework for interaction detection

Inspired by the Mango framework, we modeled the interaction probability using RE cut site density with genomic distance [21]. Interaction probability between loci $i$ and $j$ follows Bayesian formulation:


(4) \begin{equation*} P\left({I}_{ij}|{d}_{ij},{\rho}_{ij}\right)=\frac{P\left({d}_{ij},{\rho}_{ij}|{I}_{ij}\right)P\left({I}_{ij}\right)}{P\left({d}_{ij},{\rho}_{ij}\right)} \end{equation*}

where ${d}_{ij}$ represents the interaction distance and ${\rho}_{ij}$ represents RE cut site density.We confirmed the independence of distance and density (see Supplementary Methods):


(5) \begin{equation*} P\left({d}_{ij},{\rho}_{ij}|{I}_{ij}\right)=P\left({d}_{ij}|{I}_{ij}\right)P\left({\rho}_{ij}|{I}_{ij}\right) \end{equation*}

This yields:


(6) \begin{equation*} P\left({I}_{ij}|{d}_{ij},{\rho}_{ij}\right)=\frac{P\left({d}_{ij}|{I}_{ij}\right)P\left({\rho}_{ij}|{I}_{ij}\right)P\left({I}_{ij}\right)}{P\left({d}_{ij}\right)P\left({\rho}_{ij}\right)} \end{equation*}

Observed probabilities $P\left({d}_{ij}|{I}_{ij}\right)$ and $P\left({\rho}_{ij}|{I}_{ij}\right)$ represent the probability of distance and density, respectively. Combo probabilities $P\left({d}_{ij}\right)$ and $P\left({\rho}_{ij}\right)$ represent all possible anchor pairs probability of distance and density. $P\left({I}_{ij}\right)$ is determined by using the total number of anchor combos. Global correlation, per-chromosome tracking, distance stratification, and systematic subsampling uniformly reject structural collinearity. Mechanistically, orthogonal polymer physics and local biochemistry prohibit strong nonlinear coupling. Given these distinct biological dynamics, the negligible 0.2% shared variance validates our independent probability factorization (Supplementary Methods and Supplementary Fig. S4).

### Statistical significance assessment

To better handle the overdispersion problem in HiChIP data, we applied adaptive modeling. PET counts follow binomial distribution under null hypothesis:


(7) \begin{equation*} PE{T}_{ij}\sim \mathrm{Binomial}\left(N,{p}_{ij}\right) \end{equation*}

with total PETs $N$ and ${p}_{ij}$ from Equation 6.

P-value calculation:


(8) \begin{equation*} p=P\left(X\ge PE{T}_{ij}\right)=\sum_{k= PE{T}_{ij}}^N\left(\genfrac{}{}{0pt}{}{N}{k}\right){p}_{ij}^k{\left(1-{p}_{ij}\right)}^{N-k} \end{equation*}

For overdispersed data (variance-to-mean ratio > 1.5), we employ negative binomial distribution [25]:


(9) \begin{equation*} PE{T}_{ij}\sim \mathrm{NB}\left(\mu, \theta \right) \end{equation*}

Parameters $\mu$ and $\theta$ are estimated via maximum likelihood on random subsamples. When variance-to-mean ratio ≤ 1.5, binomial model suffices (Supplementary Fig. S5).

We define extreme sparse HiChIP data operationally as the condition where the unique p-value ratio, the number of distinct binomial or negative binomial p-values divided by total tested interactions, falls below 0.2. The Poisson fallback addresses conditions of extreme data sparsity in HiChIP experiments as follows:


(10) \begin{equation*} PE{T}_{ij}\sim \mathrm{Poisson}\left(\lambda \right) \end{equation*}

where $\lambda =N{p}_{ij}$.

Log-space arithmetic maintains numerical precision, and Benjamini-Hochberg correction controls FDR [24]. The default spar value of 0.35 was validated across datasets (Supplementary Figs S6–S7). Detailed descriptions of the statistical modeling procedures, including binning strategies, spline fitting, and parameter selection, are provided in Supplementary Methods.

### Experimental design and implementation

For benchmarking, we evaluated eight HiChIP analysis methods: statistical methods (sintHiChIP, FitHiChIP, HiC-DC+, MAPS, hichipper), a mix model (MMCT-Loop), and density-based clustering methods (cLoops, cLoops2). Comprehensive details on data processing, peak calling, loop calling parameters, and software versions for each method are provided in Supplementary Methods. All benchmarks were conducted on a server equipped with an AMD EPYC 7513 processor (32 cores, 64 threads) and 258 GB RAM running Ubuntu and the full hardware specifications and per-stage timing breakdowns are provided in Supplementary Methods.

### Recovery of *in situ* Hi-C and ChIA-PET loops

We obtained K562 and GM12878 HiCCUPS loops from GSE63525 [5], and GM12878 RAD21 ChIA-PET loops from Heidari et al. [26], both of which were processed by Bhattacharyya et al. [14]. We selected loops in the 20 kb to 2 Mb distance range with FDR < 0.01, ranked by FDR and then by interaction counts (descending order). For hichipper loops, we ranked by interaction counts only (PET ≥2). We computed overlap between predicted and reference loops using 5 kb tolerance.

### APA evaluation

We performed APA on HiChIP loops in the 150 kb to 1 Mb distance range. For cross-method comparison, we selected the top k loops from each method, where k equals the number of loops called by the most stringent method. We ranked loops by FDR first, then by interaction counts (descending). We ranked hichipper interactions by counts as hichipper does not provide FDR values.

We analyzed GM12878 and K562 Hi-C data using Juicer Tools (v1.6.2) with KR normalization, enforcing an identical comparative baseline across all evaluated methods [27]. We calculated P2LL and R scores directly from these KR-normalized contact intensities; this strict quantitative approach preserves absolute biological signals. To establish consistent color contrast across different datasets, APA heatmaps employed a ${\mathit{\log}}_2\left(x+1\right)$ transformation and resolution-specific min–max scaling (2nd to 98th percentile). The visual representations directly complement the rigorous quantification of underlying normalized contact frequencies.

### Benchmarking with CRISPRi-FlowFISH validation

#### Dataset preparation

We employed the K562 CRISPRi-FlowFISH and filtered 5091 pairs to 4578 enhancer-promoter pairs within the 20 kb to 1.5 Mb distance range with valid promoter-gene annotations. We classified elements as functional (showing significant CRISPRi effects) or non-functional based on experimental measurements. Following Fulco et al. [28] and Sahin et al. [15], we defined functional enhancers (also referred to as positive enhancers in HiC-DC+) as candidate regulatory elements that, upon CRISPRi perturbation, induce a significant change in target gene expression (either a decrease or a paradoxical increase, FDR < 0.05). Elements showing no significant change in expression were classified as non-functional enhancers (negative enhancers in HiC-DC+).

Loops in the 20 kb–1.5 Mb range were evaluated at four stringency levels (q < 0.01, q < 0.001, q < 1 × 10^−5^, q < 1 × 10^−7^). For hichipper (no q-values), we used equivalent PET count percentiles. A loop matched a candidate pair if anchors overlapped the enhancer and promoter region (transcription start site (TSS) ± 5 kb). When multiple loops matched, we selected the most significant loop.

#### Performance evaluation

We calculated detection rates as the percentage of candidate pairs with matching predicted loops. At the gene level, we analysed 17 genes with both functional and non-functional enhancers. Enhancers were ranked by interaction strength (−log₁₀ q-value or PET count), and precision-recall curves were computed per gene. The area under this curve, auPR, quantified enrichment of functional enhancers as follows: $auPR={\int}_0^1P(R)\mathrm{d}R$, where $P=\frac{TP}{TP+ FP}$, $R=\frac{TP}{TP+ FN}$, and the median auPR across genes was the primary metric.

At the dataset level, we evaluated all 4578 pairs. A pair was predicted positive if a significant loop (q < 0.01 or PET ≥2) overlapped both enhancer and promoter regions. Using 123 functional enhancers as ground truth, we computed: ${F}_1=\frac{2\cdotp P\cdotp R}{P+R}$.

In both gene level and dataset level analysis, TP represents detected functional enhancer numbers, FP represents detected non-functional enhancer numbers, and FN represents missed functional enhancer numbers.

### eQTL-loop regulatory integration and assessment

We integrated Genotype-Tissue Expression (GTEx) v10 eQTL data from GM12878 lymphoblastoid cells with HiChIP loops from eight analysis methods [29]. Loop coordinates were converted from hg19 to hg38 using liftOver (https://genome.ucsc.edu/cgi-bin/hgLiftOver). We filtered at q < 0.01 and ranked by q-value then interaction count. For hichipper, we used PET ≥2 and ranked by interaction count only. To ensure fair comparison, we analyzed equal numbers of top-ranked loops from each method and mapped eQTL variants (q < 0.05) to loop anchors using ±10 kb windows and required target genes to overlap the opposing anchor. We evaluated median absolute effect sizes, strong effect rates (|β| > 0.4), high significance rates (eQTL q < 1 × 10^−5^), and TSS distances. Pathway enrichment analysis was performed using Enrichr-KG (https://maayanlab.cloud/enrichr-kg) with q < 0.1 threshold, focusing on B cell receptor signaling and Epstein–Barr virus (EBV) transformation pathways relevant to GM12878 lymphoblastoid cell biology.

### Runtime and memory performance benchmarking

We measured runtime and peak memory for each sample separately and for combined samples across three HiChIP experiments, benchmarking all eight methods (sintHiChIP, FitHiChIP, HiC-DC+, MAPS, hichipper, MMCT-Loop, cLoops, cLoops2). Wall-clock time was tracked from start to finish, and peak memory usage was recorded from /proc/[pid]/status. See Supplementary Methods for details.

To isolate computational overhead, pre-computed features (e.g. binned matrices, RE density) were used for sintHiChIP and HiC-DC+. Replicates employed combined peaks for anchor standardization, ensuring fair input conditions, and focusing on execution time, while minimizing upstream variability.

## Results

### sintHiChIP demonstrates strong detection performance in Hi-C and ChIA-PET loop recovery

**Figure 2:**
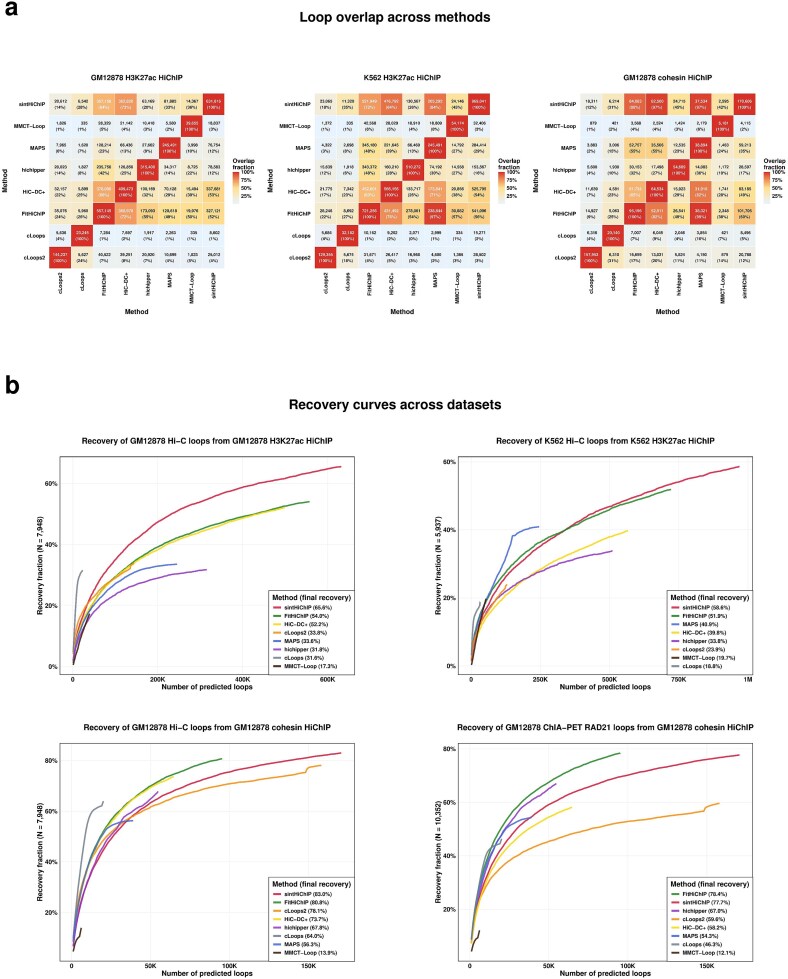
Performance of sintHiChIP loop detection in Hi-C and ChIA-PET loop recovery. Cross-method benchmarking of eight HiChIP analysis methods. (a) Pairwise overlap matrices for GM12878 H3K27ac, K562 H3K27ac, and GM12878 cohesin HiChIP (q < 0.01, PET ≥2 for hichipper). Color intensity shows concordance (red: High, yellow: Moderate, blue: Low). Diagonal shows total loops; off-diagonal shows overlap percentages. (b) Recovery curves for independent validation. Upper panels: H3K27ac HiChIP against Hi-C loops (GM12878: 7948 loops; K562: 5938 loops). Lower panels: GM12878 cohesin HiChIP validated against Hi-C loops (7948) and RAD21 ChIA-PET loops (10,352). Curves show progressive recovery ranked by statistical significance.

To assess whether sintHiChIP’s RE density modeling improves structural accuracy, we evaluated recovery of independently identified Hi-C and ChIA-PET loops. Pairwise overlap analysis revealed two distinct methodological clusters across all datasets (Fig. 2a): statistical methods (sintHiChIP, FitHiChIP, HiC-DC+, MAPS, hichipper), a mix model (MMCT-Loop) and density-based clustering methods (cLoops, cLoops2). In H3K27ac HiChIP, clustering methods showed minimal overlap with statistical approaches. In contrast, in cohesin HiChIP, both clusters maintained internal coherence but exhibited reduced separation, with clustering methods achieving moderate concordance with statistical approaches.

To quantitatively assess detection performance, we evaluated HiChIP loop recovery against independent Hi-C and ChIA-PET datasets (Fig. 2b). For GM12878 H3K27ac, sintHiChIP achieved 65.6% recovery, substantially exceeding FitHiChIP (54.0%) and HiC-DC+ (52.2%), while other methods recovered 17.3–33.8% (all *P* < 0.001). Similar patterns emerged in K562 H3K27ac: sintHiChIP recovered 58.6% versus 51.9% for FitHiChIP with remaining methods ranging from 18.8% to 40.9% (all *P* < 0.001) (Fig. 2b).

In GM12878 cohesin HiChIP, sintHiChIP recovered 83.0% of Hi-C loops, outperforming FitHiChIP (80.8%), cLoops2 (78.1%), and MMCT-Loop (13.9%, all *P* < 0.001). For RAD21 ChIA-PET loops, sintHiChIP achieved 77.7%, comparable to FitHiChIP (78.4%, *P* = 0.23) but outperforming the other methods (all *P* < 0.001) (Fig. 2b).

sintHiChIP captures P2N interactions, contributing to enhanced H3K27ac recovery. In GM12878 H3K27ac, P2N recovered 61.2% versus 37.7% for P2P, with 27.9% uniquely detected by P2N, indicating many regulatory interactions involve subthreshold ChIP signals (Supplementary Table S2). In contrast, cohesin data showed P2P dominance (80.6%) with minimal P2N contribution (2.4%).

To characterize P2N non-peak anchors, we performed chromatin state enrichment analysis (Roadmap Epigenomics 18-state ChromHMM) [30], peak genomic annotation, and ChIP-seq/ATAC-seq metaprofile analysis in both K562 and GM12878 cells. We observed a clear depletion of active regulatory states in these anchors (TssA: 0.2 times; EnhA1: 0.2–0.4 times), which were predominantly located in distal intergenic (~45%) and intronic (~35%) regions with TSS distance distributions largely resembling the genomic background (Supplementary Figs S8–S9). Metaprofile analysis showed intermediate ChIP-seq and ATAC-seq signals at anchor centers, which were markedly lower than peak regions but remained significantly higher than random genomic background levels (*P* < 0.001) (Supplementary Fig. S10).

Direct comparison of enrichment profiles confirms that P2N non-peak anchors were not equivalent to the random genomic background. They retained a higher proportion of promoter-proximal regions and showed distinct enrichment for quiescent (1.1 times), Polycomb-repressed (ReprPCWk: 1.3–1.4 times), and transcribed (Tx: 1.3–1.5 times) states. Notably, Quies enrichment was lower than the genomic background (1.1 versus 1.8–1.9), whereas ReprPC and ReprPCWk enrichment consistently exceeded background levels (1.3–1.4 times versus 0.8–1.3 times in background) (Supplementary Fig. S8). These reproducible patterns indicate that P2N non-peak anchors represent a distinct chromatin context intermediate between active regulatory elements and random genomic sequence.

### Aggregate peak analysis validates loop quality in independent Hi-C data

**Figure 3:**
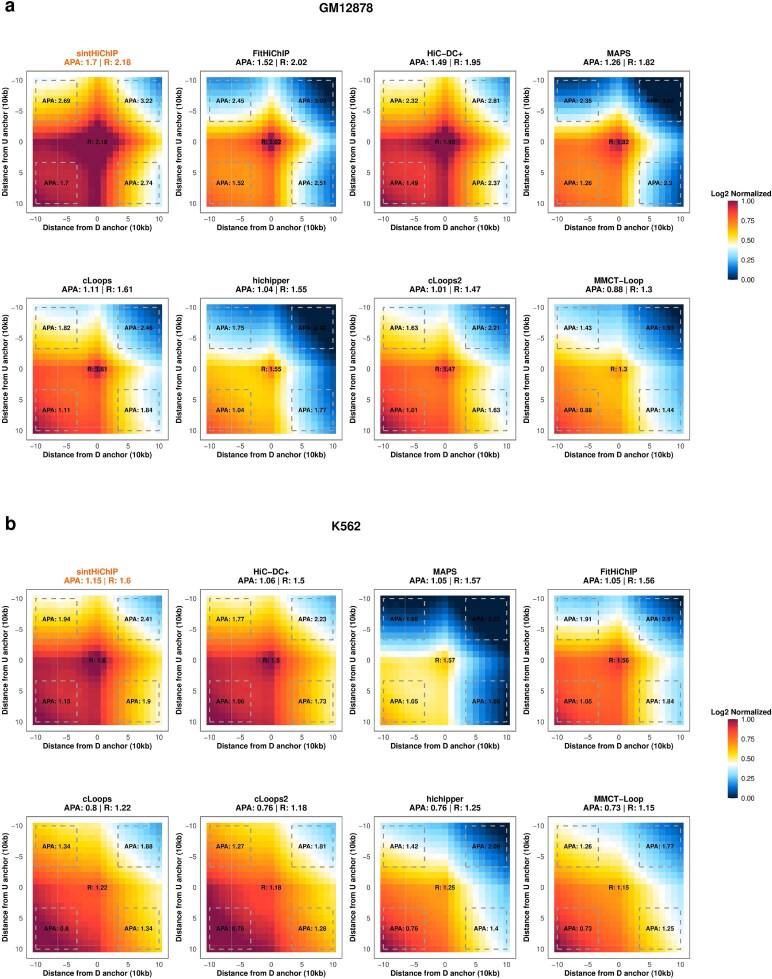
Aggregate peak analysis validates interaction quality using independent Hi-C data. Aggregate peak analysis (APA) heatmaps show contact enrichment at predicted loops across eight methods. (a) GM12878 H3K27ac (15,864 loops): sintHiChIP achieved highest enrichment (10 kb: APA 1.70). (b) K562 H3K27ac (22,929 loops): sintHiChIP maintained superior performance (APA 1.15).

To validate loop quality using independent Hi-C data, we performed aggregate peak analysis (APA) on predicted loops. APA on independent Hi-C contact matrices quantified loop enrichment using peak-to-lower-left (P2LL) ratios as APA scores and enrichment ratios (R) (Fig. 3a-b; Supplementary Table S3).

Analysis of GM12878 H3K27ac (15,864 loops, 10 kb resolution, Fig. 3a) showed that sintHiChIP achieved the highest enrichment (APA: 1.70, R: 2.18), exceeding FitHiChIP (1.52, 2.02), HiC-DC+ (1.49, 1.95), MAPS (1.26, 1.82), MMCT-Loop (0.88, 1.30), and clustering methods (1.01–1.11, 1.47–1.61). K562 H3K27ac (22,929 loops, Fig. 3b) showed consistent hierarchy: sintHiChIP (1.15, 1.60), HiC-DC+ (1.06, 1.50), FitHiChIP (1.05, 1.56), MAPS (1.04, 1.56), MMCT-Loop (0.73, 1.15), and clustering methods (0.76–0.80, 1.18–1.22). Analysis at 5 kb resolution confirmed these patterns (Supplementary Table S3). Heatmaps (Fig. 3a-b) confirmed sintHiChIP had the deepest central enrichment, with FitHiChIP and HiC-DC+ less pronounced. MAPS introduced greater heterogeneity, while hichipper, cLoops/cLoops2 showed diffuse signals.

### sintHiChIP validates functional interactions with CRISPRi-FlowFISH

**Figure 4:**
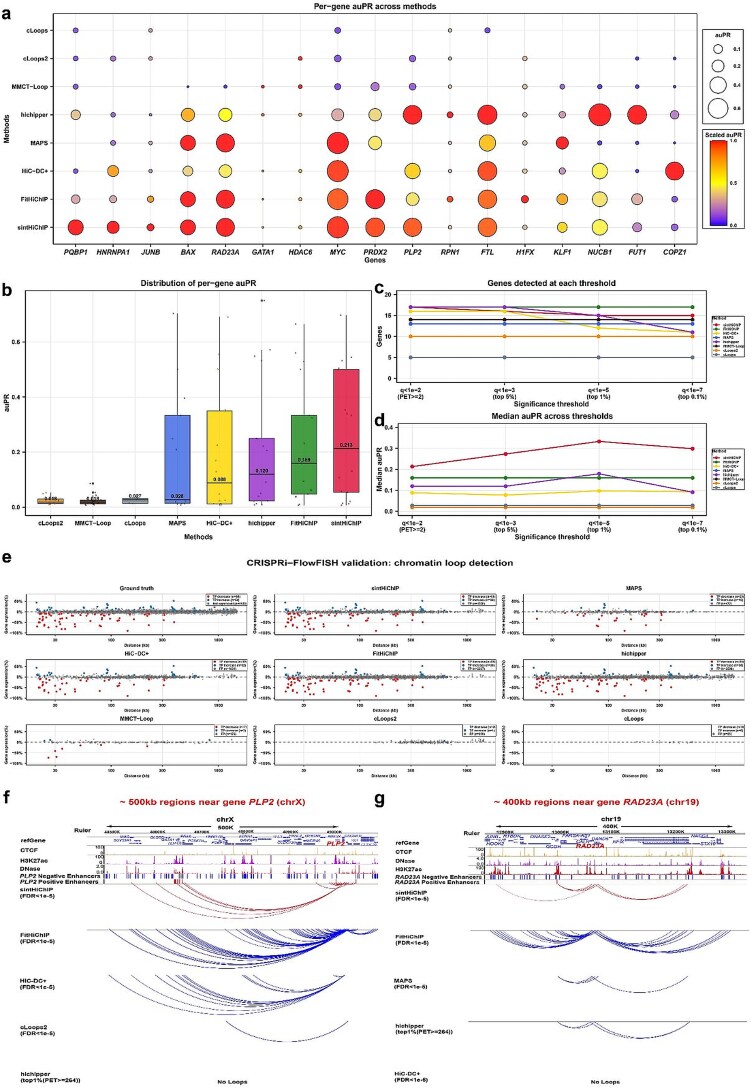
CRISPRi functional validation demonstrates enhanced regulatory interaction detection. K562 CRISPRi-FlowFISH dataset analysis. (a) Per-gene area under precision-recall curve (auPR) for 17 genes. (b) Distribution of auPR values across methods. (c) Gene-level detection completeness and (d) median auPR across multiple significance thresholds. (e) Detection patterns for all 4578 enhancer-promoter candidate pairs colored by CRISPRi classification: Red indicates functional enhancers showing expression decrease upon perturbation; blue indicates functional enhancers with paradoxical expression increase; gray indicates non-functional enhancers. (f-g) Genome browser tracks at PLP2 and RAD23A loci showing method predictions and CRISPRi-validated enhancers (red: Positive/functional enhancers, blue: Negative/non-functional enhancers).

To assess whether predicted loops represent functional interactions, we analyzed K562 H3K27ac HiChIP with CRISPRi-FlowFISH perturbation data (Fig. 4a-g). The dataset included 4578 candidate pairs with 123 enhancers exhibiting significant CRISPRi effects serving as ground truth for functional interactions.

Gene-level analysis across 17 genes (Fig. 4a) showed sintHiChIP, hichipper and FitHiChIP identified all 17, while HiC-DC+ identified 16, MMCT-Loop 14, MAPS 13, and clustering methods 5–10. Computing area under precision-recall curves (auPR, Fig. 4b), sintHiChIP achieved the highest median auPR of 0.213, followed by FitHiChIP (0.159) and other methods (0.018–0.097), with significant enrichment over HiC-DC+, MAPS, MMCT-Loop, and clustering methods cLoops and cLoops2 (*P* < 0.05).

Performance across significance thresholds revealed trade-offs in precision and sensitivity (Fig. 4c-d). At the most stringent threshold (q < 1 × 10^−7^, Fig. 4c), FitHiChIP and hichipper found all 17 genes, sintHiChIP stabilized at 15, followed by MMCT-Loop (14), MAPS (13), and HiC-DC+ (11), while clustering-based cLoops and cLoops2 identified only 5 and 10, respectively. At the commonly used threshold (q < 1 × 10^−5^, Fig. 4d), sintHiChIP demonstrated significantly better performance with auPR of 0.333, compared to 0.179 for hichipper, 0.160 for FitHiChIP, 0.089 for HiC-DC+, 0.036 for MAPS, and near-zero for MMCT-Loop, cLoops and cLoops2.

We next evaluated performance across all 4578 candidate enhancer-promoter pairs at the dataset level, assessing precision-recall trade-offs (Fig. 4e, Supplementary Fig. S11; Supplementary Table S4). Despite severe class imbalance (123 validated positives among 4578 candidates), sintHiChIP achieved the highest F1 score of 0.124 (recall: 78.9%, precision: 6.7%). While MAPS achieved slightly higher precision (7.7%, F1: 0.121), it missed over 70% of validated interactions. FitHiChIP and hichipper showed higher recall (86.2% and 83.7%) but lower precision (4.6% and 3.8%, F1: 0.087 and 0.073) due to substantially more false positives. HiC-DC+ demonstrated intermediate performance (F1: 0.116, recall: 57.7%, precision: 6.4%), while MMCT-Loop showed limited sensitivity (F1: 0.065, recall: 8.1%, precision: 5.5%), and clustering methods performed poorly (F1 scores: cLoops 0, cLoops2 0.005).

We visualized these performance differences at representative loci using Genome browser (Fig. 4f-g). sintHiChIP predictions were highly selective for CRISPRi-validated enhancers (red) over non-functional elements (blue). At the *PLP2* locus (chrX: 48.5–49.0 Mb; Fig. 4f), sintHiChIP mainly linked the promoter to validated enhancers, with few connections detected to negative elements. In contrast, FitHiChIP at comparable stringency identified numerous loops connecting to both functional and non-functional enhancers, showing higher raw sensitivity but lower specificity. The *RAD23A* locus (chr19: 12.9 Mb–13.3 Mb; Fig. 4g) exemplified this pattern, with sintHiChIP preferentially linking to validated regulatory elements, while other approaches exhibited more promiscuous connectivity. At both loci, sintHiChIP selectively identified enhancers with functional regulatory activity, excluding nearby sequences that lack measurable effects on gene expression despite similar H3K27ac enrichment, demonstrating improved precision for identifying causal regulatory interactions in disease-relevant loci.

### sintHiChIP discovers biologically coherent eQTL regulatory networks

**Figure 5:**
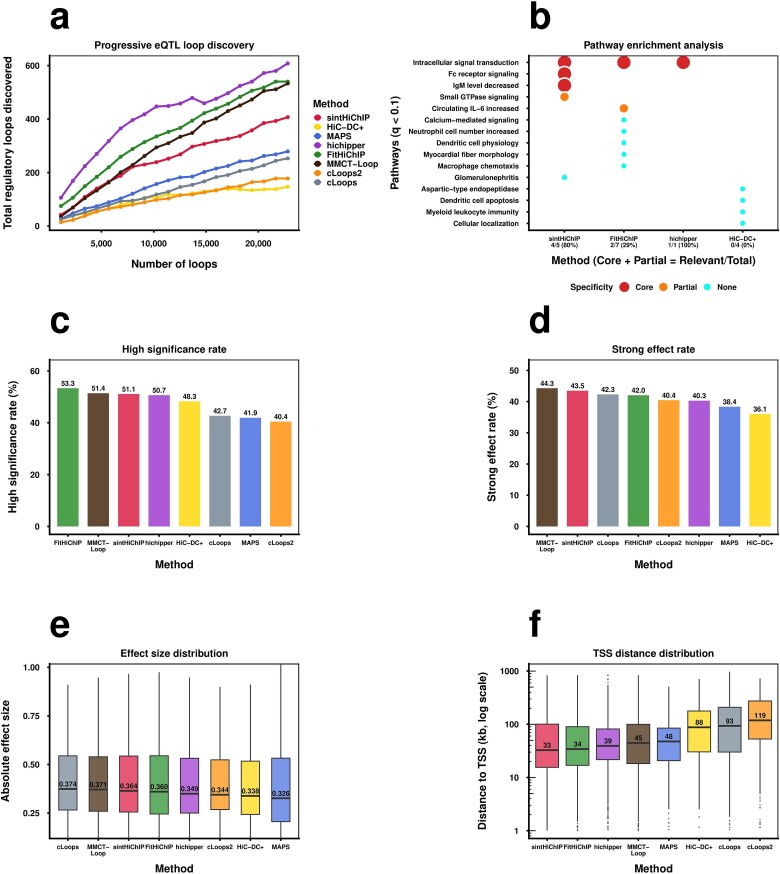
eQTL regulatory pathway analysis reveals biologically coherent networks. GM12878 eQTL-loop integration using GTEx v10. (a) Progressive discovery curves across methods. (b) sintHiChIP identifies 4 of 5 core B-cell-specific pathways (80%), including BCR signaling and Fc receptor signaling, compared to ≤2 by other methods. (c) High eQTL significance rates (proportion of eQTLs at q < 1 × 10^−5^) across methods. (d) Strong effect size rates (proportion of eQTLs with |β| > 0.4) across methods. (e) Median effect size distributions across methods. (f) Distance to transcription start site (TSS) highlighting methodological differences in promoter-proximal versus distal interaction preferences.

To explore the regulatory impact of chromatin loops, we integrated GTEx v10 eQTL data from GM12878 lymphoblastoid cells (Fig. 5). Loops were defined as interactions where eQTL variants mapped within ±10 kb of one anchor and target genes overlapped the opposing anchor (eQTL q < 0.05, 20 kb–1.5 Mb range, loop q < 0.01).

Progressive discovery curves (Fig. 5a) identified a balanced 407 regulatory loops for sintHiChIP, intermediate between higher (hichipper: 608, FitHiChIP: 540, MMCT-Loop: 533) and lower (MAPS: 279, cLoops: 253, cLoops2: 178, HiC-DC+: 147) counts.

Pathway enrichment analysis of genes connected by eQTL regulatory loops demonstrated sintHiChIP’s superior ability to identify cell-type-specific biological processes at q < 0.1 (Fig. 5b; Supplementary Table S5). Focusing on GM12878/B cell-specific pathways, sintHiChIP identified 5 pathways (q < 0.1), of which 4 (80%) were relevant to B cell biology: 3 core GM12878-specific pathways (regulation of intracellular signal transduction, Fc receptor mediated stimulatory signaling pathway, and decreased IgM level) and 1 partially related pathway (regulation of small GTPase mediated signal transduction). These pathways encompass functionally integrated B cell receptor signaling components, including key genes such as *LYN, VAV2, PLCG2* [31], as well as *TRAF3* involved in NF-κB regulation [32], characteristics of EBV-transformed lymphoblastoid cells and reflecting viral LMP2A-mediated dysregulation documented in EBV immortalization [33, 34].

By comparison, FitHiChIP detected seven pathways with two (29%) B cell-related (BCR signaling and IL-6 regulation), while five were non-specific (neutrophil, dendritic cell, myocardial pathways). hichipper identified 1 B cell pathway (BCR signaling). HiC-DC+ identified four pathways (q ≤ 0.095), none B cell-related (dendritic cell, myeloid immunity). MAPS, cLoops, cLoops2, and MMCT-Loop identified no significant pathways.

To rigorously assess the quality of these eQTL regulatory loops, we examined eQTL significance levels, effect sizes, and spatial characteristics (Supplementary Table S6). sintHiChIP achieved a very high significance rate of 51.1% (Fig. 5c, eQTL q < 1 × 10^−5^), significantly higher than MAPS (41.9%, *P* = 0.018), cLoops (42.7%, *P* = 0.035), and cLoops2 (40.5%, *P* = 0.018), with no significant difference from the remaining methods (all *P* > 0.05).

Strong effect rates (|β| > 0.4, Fig. 5d) and median effect sizes (Fig. 5e) were consistent across all methods (all *P* > 0.05), except that MAPS showed a significantly lower median effect size than sintHiChIP (0.326 versus 0.364, *P* = 0.0072).

Distance to TSS analysis evaluated both exact proximity metrics (Supplementary Table S6) and log-scaled spatial distributions (Fig. 5f). sintHiChIP (28.8 kb) and FitHiChIP (30.5 kb) exhibited the strongest promoter-proximal preference, showing no statistical difference (*P* = 0.64). hichipper (36.4 kb, *P* = 0.45), MMCT-Loop (42.4 kb, *P* = 0.21), and MAPS (43.6 kb, *P* = 0.56) followed with comparable proximal tendencies. Conversely, HiC-DC+ (72.3 kb), cLoops (84.8 kb), and cLoops2 (104.6 kb) captured significantly more distal contacts (all *P* < 0.001 versus sintHiChIP).

These metrics confirm that sintHiChIP captures consequential regulatory interactions with strong genetic effects, rigorously validating the pathway enrichment findings.

### Computational efficiency enables high-throughput analysis

**Figure 6:**
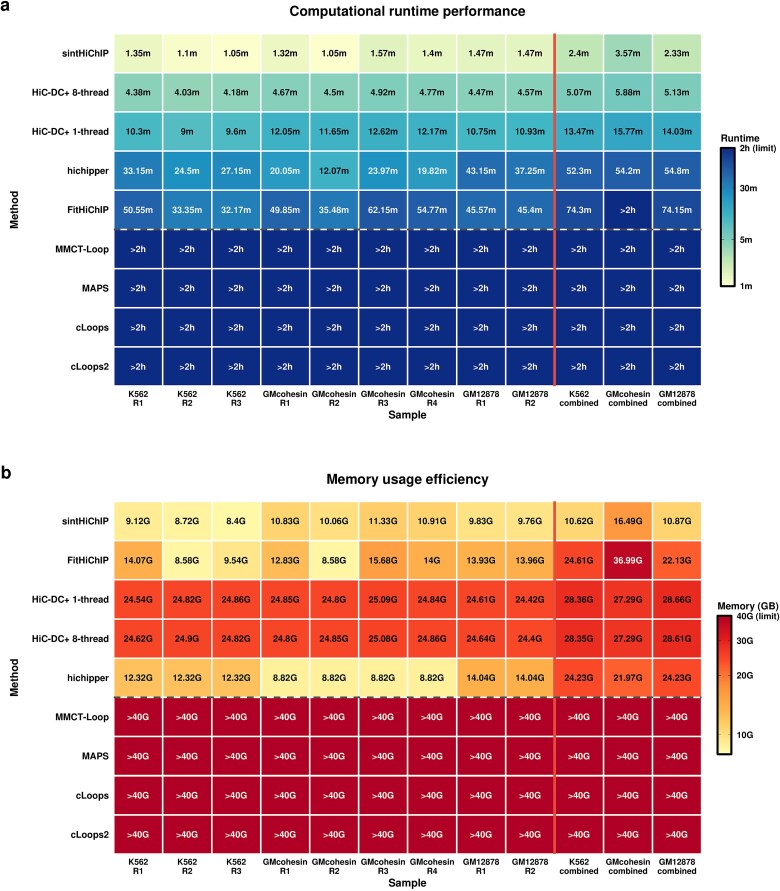
Computational performance benchmarking demonstrates substantial efficiency gains. Runtime (a) and memory usage (b) across 12 samples (9 replicates and 3 combined) from three HiChIP experiments. The dashed horizontal line separates methods completing within the benchmark thresholds (2-hour runtime, 40 GB memory) from those exceeding them; the vertical line separates individual replicates from combined samples. sintHiChIP completed replicates in ~1.05–1.57 minutes using 8.40–11.33 GB memory and combined samples in ~2.33–3.57 minutes using 10.62–16.49 GB, outperforming HiC-DC+, hichipper, FitHiChIP, and the other methods across all datasets.

To evaluate the practical scalability of sintHiChIP for large-scale studies, we benchmarked runtime and memory consumption against competing methods on 12 samples from three HiChIP datasets (Fig. 6; Supplementary Tables S7–S8). sintHiChIP achieved the fastest performance: 1.31 minutes for replicates and 2.77 minutes for combined samples, with memory usage of 9.88 and 12.66 GB, respectively. HiC-DC+ required 4.50 minutes (8-thread) or 11.01 minutes (single-thread) for replicates and 5.36 minutes (8-thread) or 14.42 minutes (single-thread) for combined samples, with ~ 24.72 and 28.09 GB memory. Hichipper required 26.79 minutes for replicates and 53.77 minutes for combined samples, with memory of 11.15 and 23.48 GB. FitHiChIP required substantially longer: 45.48 minutes for replicates and 99.77 minutes for combined samples (~35 times slower than sintHiChIP), with memory of 11.88 GB and 27.91 GB. MMCT-Loop, MAPS, cLoops, and cLoops2 exceeded the 2-hour runtime and 40 GB memory limits across all samples. Their runtimes and memory usage are recorded as >2 h and > 40G, respectively, in Supplementary Tables S7–S8 and Fig. 6. Processing stage analysis revealed computational time distribution across three stages: matrix generation/filtering (Step 1), statistical testing (Step 2), and output generation (Step 3) (Supplementary Table S9). For individual replicates, sintHiChIP exhibited relatively balanced allocation between Steps 1 and 2 (average 52% and 44% respectively), with minimal overhead for output generation (<6%). In combined samples, Step 1 increased to 62%–68% of runtime due to larger data volumes, while statistical testing remained efficient (28%–38%).

These substantial performance gains enable systematic parameter exploration and sensitivity analyses that are impractical with methods requiring 30–100 minutes per sample, particularly valuable for large-scale comparative studies across multiple conditions where dozens to hundreds of samples require analysis.

## Discussion

sintHiChIP demonstrates strong performance in identifying enhancer-promoter interactions marked by histone modifications (e.g., H3K27ac) while achieving comparable performance for cohesin loops, ranking among top-tier methods across chromatin contexts. This context-dependent advantage reflects differences in chromatin accessibility patterns: H3K27ac-marked regulatory elements exhibit variable accessibility, whereas CTCF/cohesin sites maintain uniform accessibility. APA in independent Hi-C data validated this advantage.

Unlike tools treating RE density as technical bias, sintHiChIP models it as biological information reflecting chromatin accessibility through Gaussian kernel smoothing and adaptive distribution selection based on local variance-to-mean ratios. This approach produces substantial advantages in regulatory H3K27ac HiChIP (Hi-C recovery, aggregate peak analysis, and CRISPRi validation) while achieving comparable performance to top-tier methods in structural cohesin HiChIP.

CRISPRi-FlowFISH validation provides robust independent experimental support for these findings: sintHiChIP showed superior precision in distinguishing regulatory from non-regulatory interactions, with predictions selectively targeting experimentally verified enhancers at loci such as *RAD23A* while avoiding nearby non-functional sequences. Pathway enrichment analysis of eQTL-linked regulatory networks revealed biologically coherent gene circuits specific to GM12878 lymphoblastoid cell biology, including B cell receptor signaling components (*LYN, VAV2, PLCG2*), NF-κB regulation (*TRAF3*), and characteristics of EBV-transformed cells. sintHiChIP identified more B-cell-specific pathways with higher biological coherence than competing methods, demonstrating superior detection of cell-type-specific regulatory circuits. The substantial P2N contribution in H3K27ac HiChIP reveals that many functional chromatin interactions involve anchor regions with ChIP signals below peak-calling thresholds.

Chromatin state, genomic annotation, and ChIP-seq metaprofile analyses collectively show that P2N non-peak anchors occupy a chromatin context intermediate between active regulatory elements and random genomic background. The consistency of this profile across K562 and GM12878 argues against a technical artifact explanation. The biological mechanisms underlying this distinct anchor class remain unclear.

Separate modeling of genomic distance and RE density allows the method to capture distinct chromatin forces. Folding through polymer physics and loop extrusion is reflected by distance, while biochemical states like nucleosome gaps are represented by RE density. This modeling strategy offers a perspective for future development across all proximity ligation techniques. In Hi-C, RE cut site density could similarly serve as an accessibility covariate, as RE patterns correlate with open chromatin [19]. Similarly, fragment size distributions in ChIA-PET may reflect nucleosome positioning at protein binding sites.

In summary, sintHiChIP improves detection of enhancer-promoter interactions in regulatory HiChIP data by treating RE density as a biological signal reflecting chromatin accessibility, while achieving comparable performance for structural cohesin loops dominated by uniform CTCF sites. Current implementations rely on restriction enzyme-based protocols, potentially limiting their application to enzyme-free methods like Micro-C, where alternative covariates such as MNase sensitivity would need to be explored with systematic validation. Future applications to cancer samples could identify disrupted regulatory networks where accessibility patterns shift during oncogenic transformation, while integration with ATAC-seq data would further refine chromatin state distinctions. Its computational efficiency supports both focused mechanistic studies and large-scale genomic surveys.

**Key Points**

- Modeling restriction enzyme cut site density as a biological signal enables sintHiChIP to achieve 65.6% Hi-C loop recovery in H3K27ac HiChIP (versus 17.3%–54.0% for existing methods) while maintaining a comparable 83.0% structural cohesin accuracy.
- Independent Hi-C aggregate peak analysis verifies maximum contact enrichment at sintHiChIP-predicted anchors (APA: 1.70 versus 0.88–1.52 in GM12878 H3K27ac).
- The framework establishes superior functional precision in CRISPRi-FlowFISH benchmarks (auPR: 0.213 versus 0.018–0.159) and isolates 80% of cell-type-specific regulatory pathways (versus ≤29% for other methods) across independent eQTL integrations.
- Executing over 200 million paired-end tags under 5 minutes (average 2.77 min, 12.66 GB memory) on standard workstations eliminates the computation bottlenecks of existing tools.

## Supplementary Material

Supplementary_materials_bbag292

## Data Availability

The sintHiChIP software is available at https://github.com/wding0501/sintHiChIP under the GPL-3 license. Processed HiChIP interaction files and analysis scripts are deposited in Figshare (https://doi.org/10.6084/m9.figshare.30509564).
