# Supplementary material for: Accurate and efficient HiChIP interaction detection by modeling restriction enzyme cut site density as biological signal
Source: Brief Bioinform. 2026 Jun 8;27(3):bbag292. doi: 10.1093/bib/bbag292 (PMC13245736; doi:10.1093/bib/bbag292)
Supplement: Supplementary_materials_bbag292 [file supplementary_materials_bbag292.zip › Supplementary_materials_bbag292.docx]

**Supplemental Material for “*Accurate and efficient HiChIP interaction detection by modeling restriction enzyme cut site density as biological signal”***

Weiyue Ding^1^, Yang Zhou^1^, Quanhong Liu^1^, Yiyuan Guo^2^, Chiping Zhang^1^* and Shuilin Jin^1^*

^1^ School of Mathematics, Harbin Institute of Technology, Harbin, Heilongjiang, 150001, China

^2^ Department of Ophthalmology, First Affiliated Hospital of Harbin Medical University, Harbin, Heilongjiang, 150001, China.

*Correspondence to

Chiping Zhang, zcp@hit.edu.cn;

Shuilin Jin, jinsl@hit.edu.cn

# Supplemental methods

## **Box 1: sintHiChIP pipeline overview**

sintHiChIP identifies significant chromatin interactions through five main steps:

**1. Input data processing:** Process HiChIP sequencing reads with HiC-Pro to obtain intra-chromosomal contact pairs, identify ChIP-seq peak regions, and define all peak-related interactions including both peak-to-peak (P2P) and peak-to-non-peak (P2N) anchor pairs based on peak-binned loop classification.

**2. Restriction enzyme cut site density estimation:** Map restriction enzyme (RE) cut sites genome-wide and apply Gaussian kernel smoothing to generate a continuous chromatin accessibility profile, then average densities across genomic bins.

**3. Spline probability model construction:** Build statistical models to estimate the expected interaction frequency between any two genomic loci based on their genomic distance and RE cut site density, separately accounting for both observed interactions and all possible background anchor pair combinations.

**4. Statistical significance testing:** Compare observed contact counts to expected values using appropriate statistical distributions selected automatically based on data characteristics, and apply Benjamini-Hochberg FDR correction to control for multiple testing.

**5. Significant loop output:** Report all significant chromatin loops at the specified FDR threshold (default: q < 0.01). Resulting loops naturally fall into P2P and P2N categories depending on whether both anchors or only one anchor overlaps a peak region.

**Key Innovation:** Unlike existing methods that normalize away RE cut site density as technical bias, sintHiChIP treats it as a biological signal reflecting chromatin accessibility, enabling improved detection of regulatory interactions particularly in H3K27ac HiChIP contexts.

## S1. Statistical framework details

### Equal-occupation binning strategy

Stable sampling in a statistical sense at the observed distances and densities is necessary for estimating probabilities accurately. Constant-width bins have dramatic effects on the numerical value of the bin contents in genomic data. We use equal-occupation binning to maintain constant statistical power.

Genomic distance distributions partition into k bins, each containing approximately equal observations:

$\left| \left\{ \left( i,j \right):d_{ij}\in B_{k} \right\} \right|\approx\frac{N}{K}$ (S1)

where $\left\{ d_{ij} \right\}$ represents empirical pairwise distance distribution and $\left\{ B_{k} \right\}$ denotes bin boundaries.

RE cut site densities follow identical strategy:

$\left| \left\{ \left( i,j \right):\rho_{ij}\in C_{k} \right\} \right|\approx\frac{N}{K}$ (S2)

where $\left\{ \rho_{ij} \right\}$ represents RE cut site densities.

Within each bin, interaction probabilities emerge as frequency ratios:

$P_{\text{obs}}\left( d_{k} \right)=\frac{\text{count}\left( I_{ij}\in B_{k} \right)}{N_{\text{obs}}}$ (S3)

where $N_{\text{obs}}$represents the total number of valid observed interactions across all distance bins.

### Non-parametric spline modeling

Binning produces discrete probability estimates requiring smoothing for continuous predictions. For distance bins, we calculate centers and normalized frequencies:

$\left\{ \left( x_{k},y_{k} \right) \right\}=\left\{ \left( \log_{10}d_{k},\log_{10}P_{\text{obs}}\left( d_{k} \right) \right) \right\}$ (S4)

where centers and normalized frequencies are calculated.

Smooth splines capture power-law relationships in log-space:

$f_{\text{dist}}\left( d \right)={10}^{\text{spline}\left( \log_{10}d,\left\{ x_{k},y_{k} \right\},\text{spar}=0.35 \right)}$ (S5)

### Background combo models

Appropriate null expectations require modeling all theoretically possible anchor pair combos. For each chromosome $c$, we enumerate combos within maximum interaction distance:

$\mathcal{C}_{c}=\left\{ \left( i,j \right):i,j\in\text{Anchors}_{c},\left| i-j \right|\leq d_{\max} \right\}$ (S6)

where $d_{\max}$ typically equals 2 Mb, the practical detection limit for chromatin interactions.

Total possible combos across chromosomes:

|  | $\mathcal{C}_{\text{total}}=\underset{c}{\bigcup}\mathcal{C}_{c}$ | (S7) |
| --- | --- | --- |

Background distributions follow analogous spline fitting procedures (S8-S11).

### Range clamping and boundary enforcement

Extrapolation beyond training ranges produces unreliable predictions. Valid prediction ranges are:

$d\in\left[ d_{\min},d_{\max} \right], \rho\in\left[ \rho_{\min},\rho_{\max} \right]$ (S8)

Input values clamp to these ranges before evaluation:

Spline interpolation occasionally produces predictions outside observed probability ranges. We enforce hard constraints:

$P_{\text{final}}\left( x \right)=max\left( P_{\min},min\left( P_{\text{spline}}\left( x \right),P_{\max} \right) \right)$ (S9)

Final interaction probability combines four components:

$P\left( I_{ij} \right)=\frac{P_{\text{obs}}\left( d_{ij} \right)\cdot P_{\text{obs}}\left( \rho_{ij} \right)}{P_{\text{combo}}\left( d_{ij} \right)\cdot P_{\text{combo}}\left( \rho_{ij} \right)\cdot N_{\text{total}}}$ (S10)

As empirically validated in Section S3, we approximate genomic distance and restriction enzyme density as two independent variables in our Bayesian framework (Equation 6) as follows:

$P\left( d_{ij},\rho_{ij}|I_{ij} \right)\approx P\left( d_{ij}|I_{ij} \right)\cdot P\left( \rho_{ij}|I_{ij} \right)$ (S11)

where the joint probability is factorized.

## S2. Distribution selection and p-value calculation

### Overdispersion assessment

Genomic data are frequently overdispersed relative to Poisson (or binomial) expectations. We measure this by variance decomposition:

$\phi=\frac{\text{Var}\left( X \right)}{\text{E}\left[ X \right]}$ (S12)

where $\phi>1.5$ values indicate a significant overdispersion that cannot be modeled by others without other kind of approaches. This 1.5 threshold is empirically validated in Section S3.

### Negative binomial model implementation

For data that are overdispersed, negative binomial models can be used to model the extra-Poisson variation. Maximum-likelihood parameter estimation on random subsamples (usually 10,000 interactions) is performed to achieve a good tradeoff between running time and accuracy:

$\left( \hat{\mu},\hat{\theta} \right)=arg\max_{\mu,\theta}\mathcal{L}\left( \mu,\theta|\{X_{i}{\}}_{i=1}^{n} \right)$ (S13)

Lower bounds prevent numerical instabilities:$\mu\geq1\times{10}^{-6}, \theta\geq1\times{10}^{-6}$.

P-value calculation employs log-space arithmetic handling extreme probabilities and prevent numerical underflow:

$p = \sum_{k=x}^{\infty} P_{NB\left( k;\mu,\theta\right)}, p \geq\varepsilon_{machine}$(S14)

where $\epsilon_{\text{machine}}$ represents smallest representable positive number in double precision.

### Poisson approximation fallback

When discrete distributions produce insufficient p-value resolution (unique p-value ratio < 0.2), we switch to Poisson approximation:

$X\sim\text{Poisson}\left( \lambda\right), \lambda=N\cdot P\left( I_{ij} \right)$ (S15)

P-value ratio threshold: When data sparsity is extreme, most interactions have near-zero counts, causing a large fraction of Binomial p-values to collapse to 1 and lose discriminative resolution. We define this condition as a unique p-value ratio below 0.2. Under such conditions, sintHiChIP switches to a Poisson model as a fallback, which provides a smooth and discriminative p-value distribution where Binomial cannot. This threshold is an operational definition of extreme sparsity and is described in the main Methods section; the Poisson fallback is retained as a defensive safety net for non-standard or extremely low-depth libraries.

## S3. Validation of statistical assumptions

### Validation of RE cut site density

To validate that RE cut site density reflects chromatin accessibility rather than technical bias, we performed stratified enrichment analysis across genomic regions with varying RE density. The genome was partitioned into 5 kb bins using identical parameters as sintHiChIP loop calling. RE density for each bin was calculated using Gaussian kernel smoothing (Equation 1). All observed interactions and all possible anchor pair combinations within the 20 kb–2 Mb distance range were independently stratified into 10 equal-occupation deciles based on joint RE cut site density (the product of the two anchor densities).

For each decile $k$, we calculated the site gain factor as the ratio of observed to expected interaction probability:

$\text{gain}(k)=\frac{P_{\text{obs}}(k)}{P_{\text{null}}(k)}$ (S16)

where $P_{\text{obs}}(k)$represents the empirical probability that observed interaction anchors fall within decile $k$, computed as the fraction of all interactions whose joint RE density falls in that decile. $P_{\text{null}}(k)$represents the expected probability under the null model, defined as the proportion of all possible anchor pair combinations within the 20 kb–2 Mb distance range whose joint RE density falls within decile $k$. This null model enumerates the complete set of genomic anchor pair combinations, providing a more stringent background than random genomic sampling by explicitly accounting for the genomic distribution of all potential interactions. A site gain factor $>1$ indicates enrichment of interactions in regions of that RE density level relative to the genomic null expectation; a factor $\left< 1 \right.$ indicates depletion.

We applied this analysis to GM12878 H3K27ac and GM12878 cohesin HiChIP datasets. H3K27ac HiChIP showed strong monotonic enrichment across deciles with a median site gain of 2.40 (range: 0.05–24.1), whereas cohesin HiChIP exhibited substantially lower enrichment (median: 1.24, range: 0.26–2.68). The significant difference between regulatory and structural contexts (Wilcoxon rank-sum test, p < 2.2×10⁻¹⁶) and the distinct dynamic ranges are inconsistent with uniform technical bias, confirming that RE cut site density encodes chromatin accessibility information that differs between regulatory and structural chromatin contexts (Supplemental Figs. S2–S3).

### Empirical validation of independence assumption

To validate the independence assumption between genomic distance (loopWidth) and joint RE cut site density, we performed four complementary analyses. (1) Global Pearson correlation: cor.test() was applied to loopWidth and log2(joint cut site density) on the full GM12878 H3K27ac dataset (n = 16,202,389), yielding R², VIF = 1/(1 − R²), and p-value. (2) Per-chromosome analysis: the same correlation was computed independently for each chromosome to test whether any single chromosomal region drives the global result. (3) Distance-stratified analysis: interactions were partitioned into four distance bins (20–500 kb, 500 kb–1 Mb, 1–1.5 Mb, 1.5–2 Mb) and Pearson R² was computed within each stratum to directly assess conditional independence across genomic scales. (4) Subsampling validation: 100 independent random subsamples without replacement were drawn at six sample sizes (n = 100,000; 200,000; 500,000; 1,000,000; 2,000,000; 5,000,000) across all three HiChIP datasets, with R², VIF, and −log10(p) computed per replicate (Supplemental Fig. S4).

We empirically validated the independence between genomic distance and joint cut site density using GM12878 H3K27ac HiChIP data (n = 16,202,389 interactions). Pearson correlation analysis yielded R² = 0.00199 (p < 2.2×10⁻¹⁶), indicating that genomic distance explains only 0.2% of the variance in joint cut site density. Variance Inflation Factor (VIF = 1.002), calculated as VIF = 1/(1-R²), was substantially below the multicollinearity threshold (VIF < 5), confirming negligible collinearity between these covariates.

Per-chromosome analysis showed consistent low correlations (mean R² = 0.004465, range: 0.000075-0.023477 across all chromosomes). Stratification by distance bins (20-500 kb, 500 kb-1 Mb, 1-1.5 Mb, 1.5-2 Mb) yielded similarly low R² values (all < 0.01), demonstrating that independence holds across different genomic scales.

We acknowledge that the combination of small R² and significant p-value (p < 2.2×10⁻¹⁶) warrants clarification. These two statistics measure fundamentally different properties: R² quantifies effect size (the proportion of shared variance), while p-value reflects statistical power, which scales with sample size regardless of effect size.

To rigorously assess whether any true correlation exists between loopWidth and RE site density, we performed subsampling validation across all three datasets at six sample sizes (n = 100,000 to full dataset, 100 replicates each), computing Pearson R² and VIF at each level (Supplemental Fig. S4).

The key diagnostic is the trajectory of R² as n increases:

- **GM12878 cohesin** (n = 15,482,273): Full-dataset R² = 0.0004%, VIF = 1.000004. R² decreased monotonically as n increased, while −log₁₀(p) rose from near zero to ~40. This divergence — R² converging toward zero while significance increases — is the definitive statistical signature of a null effect driven purely by power, not by any true correlation signal.
- **GM12878 H3K27ac** (n = 16,202,389): Full-dataset R² = 0.199%, VIF = 1.00199. R² remained stable across all sample sizes (~0.197–0.213%), and −log₁₀(p) reached ~300 at full n. Although this dataset shows the highest R², 0.199% means the two covariates share less than 0.2% of variance — a biologically negligible association.
- **K562 H3K27ac** (n = 40,378,771): Full-dataset R² = 0.016%, VIF = 1.000159. R² decreased from 0.026% at n = 100,000 to 0.016% at n = 5,000,000, consistent with the null convergence pattern.

The empirical validation (Supplemental Fig. S4) clarifies the divergence between effect size and statistical significance. As shown in panel a, the Pearson R² remains consistently near zero (≤ 0.2%) across all sample sizes, indicating a negligible association. Meanwhile, panel c demonstrates that the extreme p-value (p < 2.2 × 10⁻¹⁶) is a direct consequence of increasing sample size rather than a reflection of true biological dependency. With VIF values stable at ≈ 1 (panel b), these results robustly justify treating genomic distance and RE density as independent covariates in the sintHiChIP model.

Furthermore, even under the conservative assumption that the observed correlation is real rather than a power artifact, the impact of any residual dependence on the factorization in Equation 5 is negligible in practice. The joint probability can be written as:

$P(d,\rho\mid I)=P(d\mid I)\cdot P(\rho\mid I)\cdot(1+\epsilon)$ (S17)

where the error term $\epsilon$represents the deviation from strict independence. The magnitude of $\epsilon$is directly determined by the degree of association between $d$and $\rho$, which is empirically quantified by the Pearson correlation coefficient $r$ and the variance inflation factor (VIF). The observed $R^{2}=0.00199$ ($r\approx0.045$) and $\text{VIF}=1.002$ indicate that genomic distance and joint RE cut site density share less than 0.2% of variance. This near-zero covariance confirms that $\epsilon\approx0$ across the full dataset, and that the factorization in Equation 5 introduces negligible bias relative to the dynamic range of interaction probabilities spanning several orders of magnitude in HiChIP data. These results support the independence assumption.

We note that low linear correlation does not strictly guarantee independence in the presence of nonlinear dependencies. However, given the biological context in which genomic distance reflects polymer physics and RE density reflects local chromatin biochemistry — two mechanistically orthogonal processes — strong nonlinear coupling between these variables is not expected. The independence approximation in Equation 5 is therefore statistically justified.

### Simulation-based validation of the dispersion threshold

sintHiChIP employs an adaptive distribution selection strategy, utilizing a variance-to-mean ratio > 1.5 as the default threshold to switch from a Binomial to a Negative Binomial (NB) model. We validated this threshold through two simulation experiments (Supplemental Fig. S5).

**Dispersion threshold validation:** Expected interaction probabilities (μ) for each interaction pair were derived from each of the three real HiChIP datasets (GM12878 H3K27ac, K562 H3K27ac, GM12878 cohesin) using the fitted sintHiChIP spline models. Synthetic PET count datasets were simulated across 11 target variance-to-mean ratios (φ = 1.0, 1.1, 1.2, 1.3, 1.5, 1.7, 2.0, 2.5, 3.0, 5.0, 10.0). In each simulation, 2% of interactions were randomly designated as true positives at two enrichment levels (2 times and 5 times over expected background μ); the remaining 98% served as true negatives. Five independent replicates were generated per condition using unique seeds. Significant interactions were called at FDR < 0.01 using binomial, NB, and Poisson models, and F1 scores were computed against the known true positive labels. The NB advantage threshold was defined as ΔF1 (NB − binomial) > 0.01.

**Model performance crossover:** By simulating datasets across a target dispersion range (1.0 to 10.0), we observed that the NB model begins to consistently outperform the Binomial model at a dispersion level of approximately 1.3–1.7.

**Threshold optimality:** At the default threshold of 1.5, the F1-score gain (ΔF1) of the NB model over the Binomial model exceeded 0.01 across all tested datasets. This indicates that 1.5 is the optimal transition point where the NB model effectively captures the over-dispersion inherent in HiChIP data without introducing unnecessary model complexity in low-dispersion scenarios.

### Sensitivity analysis of the spline smoothing parameter (spar)

The smoothing parameter *spar* in the smooth.spline function controls the trade-off between the smoothness of the background model and its goodness-of-fit. To justify our default choice of *spar* = 0.35, we systematically evaluated 20 different *spar* values (ranging from 0.05 to 1.0) across three benchmark datasets (Supplemental Fig. S6).

**Stability of recovery and loop counts:** In all datasets, both the number of identified significant loops and the Hi-C loop recovery rate remained highly stable within the *spar* range of 0.1 to 0.45. For instance, in the GM12878 H3K27ac dataset, the variation in loop counts was less than 1.8%, and the Hi-C recovery rate fluctuated by less than 0.6 percentage points. When *spar* > 0.5, we observed a rapid inflation in loop counts, likely due to over-smoothing of the background model leading to increased false positives.

**Model fitting assessment (RSS):** Notably, Supplemental Fig. S7 illustrates the relationship between model fitness (RSS) and smoothing intensity on a Log₁₀ scale, showing a remarkable convergence across distinct chromatin architectures. At our default of 0.35 (red dashed line), the average RSS fold change remains moderate (≈33 times across datasets, log₁₀ ≈ 1.5), well below two orders of magnitude relative to the baseline. However, beyond the threshold of spar = 0.50, RSS increases sharply to approximately three orders of magnitude and continues to escalate, exceeding four orders of magnitude by spar = 0.60 (Supplemental Fig. S7).

**Conclusion:** Quantitative evaluations confirm that model performance remains stable across three biologically distinct datasets (two regulatory H3K27ac and one structural cohesin) within the wide spar = 0.1–0.45 range; the default value of 0.35 thus provides reliable genome-wide modeling without requiring dataset-specific parameter tuning.

## S4. Data Processing

### HiChIP data pre-processing

We aligned pooled HiChIP reads to hg19 reference assemblies using HiC-Pro (v3.1.0) [1], which removed PCR duplicates and invalid ligation products following FitHiChIP [2] pipeline settings.

### HiChIP peak calling

We obtained pre-called HiChIP peaks for GM12878 H3K27ac, K562 H3K27ac, and GM12878 cohesin datasets from Bhattacharyya et al. [2]. These peaks were originally called from multiple HiChIP read categories including dangling end, self-cycle, re-ligation, and cis short-range valid reads (<1 kb) following duplicate removal, directly from FitHiChIP peak calling criteria. When using hichipper to call individual peaks, we set the (EACH,SELF) option, which uses adapted MACS for peak calling [3].

### HiChIP loop calling

We obtained published loop calls for GM12878 H3K27ac, K562 H3K27ac, and GM12878 cohesin from the original publications: FitHiChIP loops from Bhattacharyya et al. [2] (GM12878 H3K27ac: Table_GH-ALL; K562 H3K27ac: Table_KH-ALL; GM12878 cohesin: Table_GC-ALL), MAPS loops from Juric et al. [4], and hichipper loops from Lareau and Aryee [5].

We ran the faster method with the following parameters on individual and combined replicates:

- - - - **hichipper**: We ran hichipper with parameters --min-dist 20000 --max-dist 2000000 --skip-diffloop --make-ucsc --keep-temp-files.
- **FitHiChIP**: We configured IntType = 3, BINSIZE = 5000, LowDistThr = 20000, UppDistThr = 2000000, UseP2PBackgrnd = 0 (loose background model to remain consistent with other peak-to-all methods that model background using all interaction types), BiasType = 1, MergeInt = 0 (no merge filtering, consistent with other methods tested), QVALUE = 0.01.
- **sintHiChIP**: We ran sintHiChIP with FDR < 0.01 and 20 kb to 2 Mb distance range.
- **HiC-DC+**: we processed data with uniform 5 kb binning (bin_type=“Bins-uniform”) and default loop identification parameters [6]. We tested the time with memory with single thread and 8 threads, respectively.

We ran the time-consuming methods with the following specific parameters. MMCT-Loop was run with relaxed parameters (-cpu 40, -minPts 20, -M 32, -ef 500, -k 4, -bs 3) on individual and combined replicates to achieve completion within practical time limits [7]. We tested MAPS using the same inferred peaks as FitHiChIP and sintHiChIP, applying the following parameters: bin_size = 5000; fdr = 2; filter_file = "None"; generate_hic = 0; mapq = 30; length_cutoff = 1000; threads = 40; per_chr = 'True'; BINNING_RANGE = 2000000. For cLoops and cLoops2, we converted allValidPairs files to BEDPE format and processed them using 40 threads with parameters -eps 2500,5000, -minPts 10, and -hic [8, 9].

### Distance range justification

The default analysis range of 20 kb to 2 Mb is justified as follows:

**Lower bound (20 kb):** Interactions below 20 kb are dominated by self-ligation artifacts and cannot be reliably distinguished from random proximity contacts at 5–10 kb bin resolution. Therefore, a 20 kb cutoff is necessary to ensure high-confidence loop calling, which is consistent with the default lower bounds widely applied in mainstream HiChIP methods (e.g., FitHiChIP).

**Upper bound (2 Mb):** Methodologically, contact frequencies beyond 2 Mb decay to extreme sparsity, approaching uniform background noise. Functional enhancer–promoter interactions are topologically constrained and predominantly occur within local chromatin domains that fall within this range [10, 16]. These parameters are user-configurable for specific biological questions.

## S5. Computational performance and efficiency benchmark

Computational environment and datasets

All computational performance evaluations were conducted on an Ubuntu server equipped with an AMD EPYC 7513 processor (32 cores, 64 threads), 258 GB RAM, and 1.8 TB NVMe storage. Benchmarking was performed across 9 individual replicates (3 K562 H3K27ac, 4 GM12878 cohesin, and 2 GM12878 H3K27ac) and 3 deeply sequenced combined datasets: GM12878 H3K27ac (185M filtered intra-chromosomal PETs), K562 H3K27ac (174M PETs), and GM12878 cohesin (153M PETs). Full runtime and memory details of the comparison methods for all datasets are provided in Supplemental Tables S7-S8.

sintHiChIP processing stage breakdown and runtimes

The sintHiChIP processing pipeline is divided into three main stages with distinct computational characteristics: (1) matrix generation and peak filtering, (2) statistical significance testing, and (3) browser track generation.

For individual replicates, the total runtime ranged from 62.6 to 94.0 seconds. Specifically, Step 1 required 25.9–52.1 seconds (41.4–59.2% of the total time), Step 2 took 26.5–44.1 seconds (38.2–52.9%), and Step 3 took 0.8–3.6 seconds (0.9–5.7%).

For the deeply sequenced combined samples, the total runtime ranged from 140.0 to 214.0 seconds, distributed as follows: 92.0–131.5 seconds for Step 1 (61.4–67.6%), 39.8–80.2 seconds for Step 2 (27.6–37.5%), and 2.3–8.2 seconds for Step 3 (1.1–5.9%). Full runtime details for all datasets are provided in Supplemental Table S9.

Computational bottleneck analysis

Overall, sintHiChIP exhibits a balanced computational distribution between matrix operations and statistical testing. This algorithmic architecture scales evenly with dataset complexity and is highly suited for parallel processing environments, ensuring robust efficiency even when processing massive HiChIP datasets.

## S6. CRISPRi validation details

### K562 dataset processing

K562 CRISPRi-FlowFISH data [11] contained 5,091 enhancer-promoter pairs. We finally obtained 4,578 pairs within 20 kb to 1.5 Mb distance range with valid gene annotations. Classification as functional required significant CRISPRi effect (adjusted p-value < 0.05 and absolute effect size > 0). This yielded 123 functional pairs (2.7% of total), creating severely imbalanced classification task.

### Loop matching criteria

We matched predicted loops to candidate pairs using the following criteria: - Enhancer region defined as start-end coordinates (no additional tolerance) - Promoter region defined as TSS ± 5 kb - Both anchor orientations tested (enhancer-promoter and promoter-enhancer) - Best match selected by lowest q-value (or highest PET count for hichipper)

### Performance metric selection

Severe class imbalance (2.7% functional pairs) renders accuracy and standard precision-recall metrics misleading. Area under precision-recall curve (auPR) provides more appropriate measure for imbalanced datasets, focusing on performance with positive class while accounting for varying decision thresholds.

F1 scores capture precision-recall trade-offs at single threshold (q < 0.01). Methods with high recall but low precision (many predictions including many false positives) achieve lower F1 than methods balancing both metrics. This penalizes “predict everything” strategies lacking discriminative power.

### hichipper threshold mapping

hichipper does not provide q-values. We established equivalent stringency levels using percentile-based PET thresholds calculated from loops with PET ≥ 2: - Baseline: PET ≥ 2 (corresponding to q < 0.01) - Top 5%: 95th percentile (corresponding to q < 0.001) - Top 1%: 99th percentile (corresponding to q < 1×10⁻⁵) - Top 0.1%: 99.9th percentile (corresponding to q < 1×10⁻⁷)

This approach ensures fair comparison while respecting methodological differences across tools.

## S7. Anchor characterization

P2N anchor properties were characterized using three complementary approaches: ChromHMM chromatin state enrichment analysis, genomic feature annotation, and ChIP-seq/ATAC-seq metaprofile analysis, applied to both K562 and GM12878 cell lines.

To establish a rigorous negative control, the genomic background was defined as randomly sampled 5 kb tiles explicitly depleted of known regulatory elements. We systematically excluded: (1) all interaction anchors (P2P, P2N peak, P2N non-peak); (2) H3K27ac peak regions; and (3) promoter-proximal zones (TSS ± 2 kb).

ChromHMM chromatin state enrichment. Chromatin state annotations were obtained from the Roadmap Epigenomics 18-state ChromHMM model (K562: E123; GM12878: E116) [12], downloaded from the WashU Epigenome Browser. For each anchor group, observed chromatin state distributions were computed by intersecting anchor BED files with ChromHMM annotation files using bedtools (v2.30.0) intersect [13]. To assess enrichment versus random expectation, 100 size-matched genomic shuffles were generated for each anchor group using bedtools shuffle -noOverlapping -seed i against hg19 chromosome sizes. Log2 fold enrichment was computed as log2(observed state frequency / mean shuffled state frequency).

Genomic feature annotation. Genomic features were assigned using ChIPseeker (v1.28.0) [14] with GENCODE v19 annotations. Anchors were categorized as promoter (±1–3 kb from TSS), 5’ UTR, 3’ UTR, exon, intron, downstream, or distal intergenic; TSS distances were calculated bidirectionally to the nearest transcription start site.

ChIP-seq and ATAC-seq metaprofile analysis. CTCF, RAD21, SMC3, and ATAC-seq signals were extracted using deepTools (v3.5.2) with computeMatrix reference-point mode (±5 kb windows centered on anchor midpoints, bin size = 50 bp) [15]. This was applied independently to each anchor group and the genomic background. Mean signal profiles were plotted using plotProfile.

Results. P2P peak and P2N peak anchors exhibited strong enrichment for active regulatory chromatin states in both cell lines (TssA: 3.1–3.9 times; EnhA1: 2.6–3.0 times; TssFlnkU: 3.1–3.6 times) and localized predominantly to promoter-proximal regions, accompanied by high CTCF, RAD21, SMC3, and ATAC-seq signals at anchor centers. P2N non-peak anchors demonstrated marked depletion of all active states (TssA: 0.2 times; EnhA1: 0.2–0.4 times) and were primarily situated in distal intergenic and intronic regions. However, these sub-threshold anchors did not collapse into the random background. They projected intermediate enrichment for transcribed (Tx: 1.3–1.5 times), Polycomb-repressed (ReprPCWk: 1.3–1.4 times), and quiescent (Quies: 1.1 times) states, while retaining a measurable spatial bias toward promoter-proximal regions relative to the background control. Statistical evaluation confirmed that P2N non-peak anchors consistently maintained a significant signal elevation above the random genomic baseline across all tested architectural and regulatory markers in both K562 and GM12878 cell lines (p < 0.001, Wilcoxon rank-sum tests). This cross-cell-line consistency indicates that P2N non-peak loci constitute a biologically distinct chromatin state rather than technical artifact (Supplemental Figs. S8-S10).

## S8. Dataset details

Published sources: GM12878 and K562 H3K27ac HiChIP data (GSE101498, SRR5831489–SRR5831493) [16], GM12878 cohesin HiChIP data (GSE80820, SRR3467175 - 3467178) [10], GM12878 and K562 Hi-C data (GSE63525) [17], K562 CRISPRi-FlowFISH data [11], GTEx v10 eQTL data (<https://gtexportal.org>) [18], and Hi-C HiCCUPS loops for GM12878 and K562 [16] and GM12878 RAD21 ChIA-PET loops [19] were obtained through processed files from Bhattacharyya et al. [2] for benchmarking.

The ChIP-seq tracks, visualized using WashU genome browser (https://epigenomegateway.wustl.edu/browser2022/) or ChIP-seq coverage analysis, were downloaded from Encyclopedia of DNA Elements (ENCODE) project [20] (https://www.encodeproject.org; hg19) and GEO as follows:

**K562**

- CTCF (ENCFF000BWF): https://www.encodeproject.org/files/ENCFF000BWF
- H3K27ac (ENCFF000BWY): https://www.encodeproject.org/files/ENCFF000BWY
- DNase (ENCFF526LYS): https://www.encodeproject.org/files/ENCFF526LYS
- RAD21 (ENCFF000YXZ): https://www.encodeproject.org/files/ENCFF000YXZ
- SMC3 (ENCFF000YZM): https://www.encodeproject.org/files/ENCFF000YZM
- ATAC-seq: GEO sample number: GSM2695560

**GM12878**

- CTCF (ENCFF000ROP): https://www.encodeproject.org/files/ENCFF000ROP
- RAD21: GEO sample number: GSM935332
- SMC3: GEO sample number: GSM935376
- ATAC-seq: GEO sample number: GSM7854725

## S9. RE cut site density file generation

The normsite.R script normalizes MboI RE cut site density across the hg19 genome in 5 kb bins with a Gaussian kernel (variance = 100,000), aggregating information to access HiC-DC+’s precomputed features for efficient chromatin interaction modeling in sintHiChIP. At ~33 seconds (output: 25.03 MB), this rapid step does not block the full flow, enabling processing of large HiChIP datasets (e.g., 205M pairs for K562 H3K27ac). Taking RE density as a biological signal for chromatin accessibility (see main Methods, Equation 1–3), the normsite.R file enhances sintHiChIP’s interaction probability modeling, improving accuracy over HiC-DC+’s normalization approach, thus explaining part of its strong performance (e.g., 65.6% Hi-C loop recovery in GM12878 H3K27ac). The file is produced through the sintHiChIP pipeline with parameters including a 20 kb to 2 Mb distance range, FDR threshold of 0.01, and hg19 reference genome (https://github.com/wding0501/sintHiChIP), ensuring reproducibility and adaptability for customized analyses. Method-specific parameters are detailed in the main Methods section.

## S10. Statistical analysis

Statistical comparisons across methods were conducted for loop recovery rates (Figure 2), functional validation (Figure 4), and eQTL-regulatory loop quality metrics (Figure 5). For categorical outcomes, including loop recovery rates and proportions meeting significance or effect size thresholds, chi-square tests were applied to 2×2 contingency tables without continuity correction (chisq.test(..., correct = FALSE) in R). Fisher’s exact test was used when expected cell counts were < 5 (e.g., gene-level detection in Figure 4a). For continuous or ordinal outcomes, such as effect size magnitudes, TSS distances, and per-gene auPR values, two-sided Wilcoxon rank-sum tests were employed (wilcox.test(..., exact = FALSE)). Paired Wilcoxon signed-rank tests (paired = TRUE) were used for auPR comparisons across the same genes. Significance was assessed at α = 0.05, with p-values reported individually without multiple testing correction. P-values that smaller than 2.2×10⁻¹⁶ are denoted as p < 2.2×10⁻¹⁶. All analyses were performed in R version 4.3.2 using base functions and the PRROC package [21].

## S11. Software versions and parameters

All analyses employed the following software versions:

- sintHiChIP: version 1.0.0
- FitHiChIP: v8.1.0
- HiC-DC+: v0.2.2
- MAPS: v1.1.0
- hichipper: v1.1.0
- cLoops: v0.92
- cLoops2: v0.0.3
- MMCT-Loop: v1.0.0
- HiC-Pro: v3.1.0
- Juicer Tools: v1.6.2
- deepTools: v3.5.2
- bedtools: v2.30.0
- R: v4.3.2

Common parameters across all methods: - Distance range: 20 kb to 2 Mb - FDR threshold: 0.01 (where applicable) - Reference genome: hg19 (human)

Method-specific parameters detailed in main Methods section.

**Note on hichipper**: hichipper processes multiple samples sequentially within a single run. For individual replicate statistics, we reported the peak memory usage (which represents the maximum memory required for the largest sample in the batch). For combined samples, hichipper was run on the combined dataset as a single sample.

## S12. Practical usage guide

RE density files must be regenerated for each restriction enzyme, as cut site distributions are enzyme-specific.

1) The sintHiChIP GitHub repository provides scripts for generating density files for common enzymes (<https://github.com/wding0501/sintHiChIP>).

2) sintHiChIP is not directly applicable to enzyme-free methods (e.g., Micro-C) without adaptation of the density estimation module.

3) sintHiChIP may not be appropriate for: a) extremely low-depth HiChIP libraries where distance bins contain insufficient interactions for robust distribution fitting; b) targeted capture HiChIP variants where genome-wide binning assumptions are violated; c) enzyme-free protocols without modification.

# Supplemental figures

**
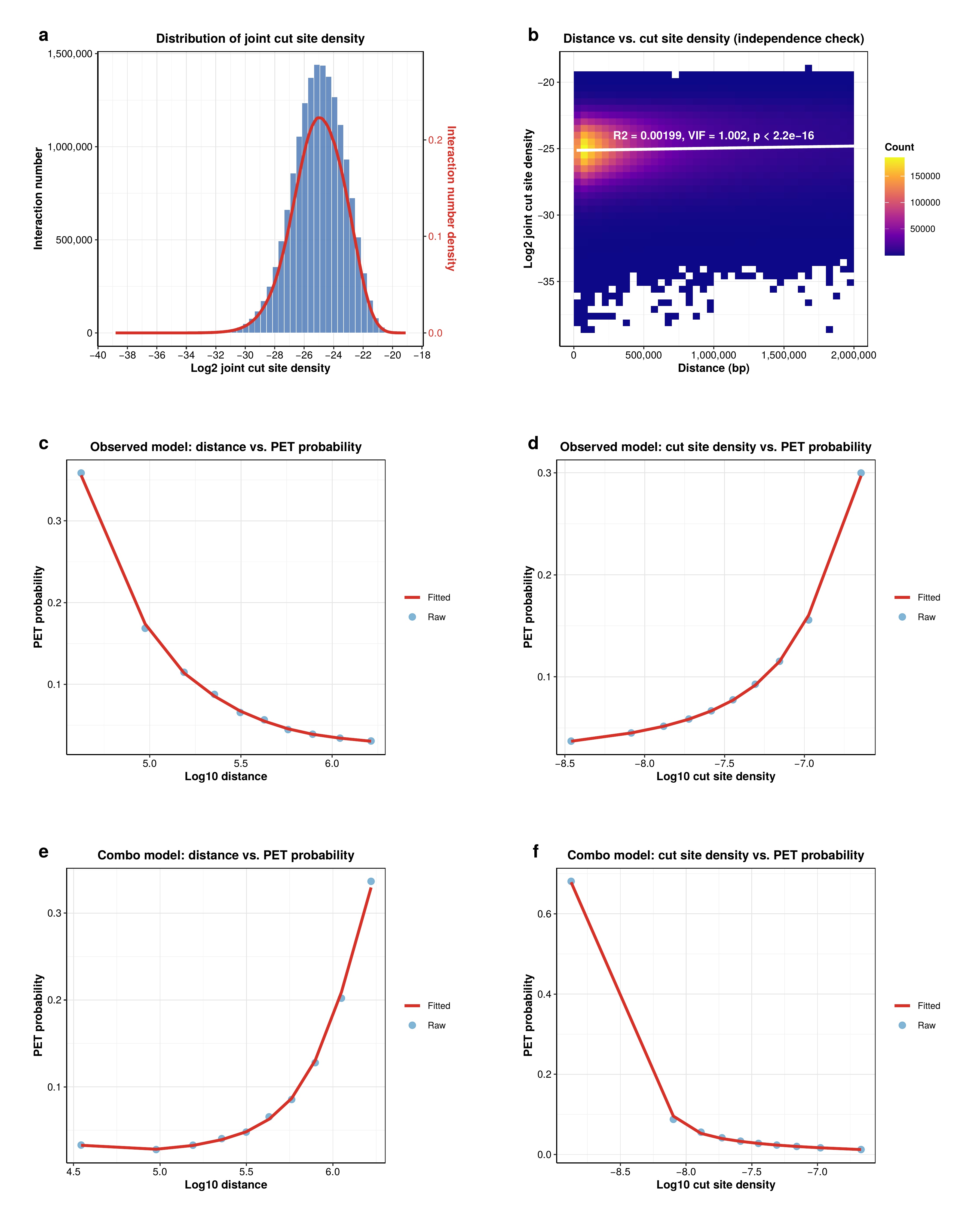
**

**Supplemental Figure S1.** Statistical framework validation and model characteristics. sintHiChIP probability model components and statistical independence verification for GM12878 H3K27ac HiChIP genome-wide analysis. (a) Distribution of log2 joint cut site density across chromatin interactions. Histogram (blue bars) shows empirical frequency distribution; red curve represents fitted kernel density estimate. Distribution exhibits bell-shaped pattern centered around -7.5, spanning approximately 3 orders of magnitude. (b) Scatter plots quantify the relationship between genomic distance and joint site density; the shared variance is strictly bounded (R² = 0.00199, p < 2.2×10⁻¹⁶, VIF = 1.002) (c, d) Observed models capture the marginal effects of individual covariates on PET probability: interaction probability decays with genomic distance (c) and increases with cut site density (d). (e, f) Combo models characterize the joint background expectation across all possible anchor pair combinations; curve trajectories are inverted relative to the observed models. Across all four panels, empirical contact frequencies tightly trace the fitted splines.


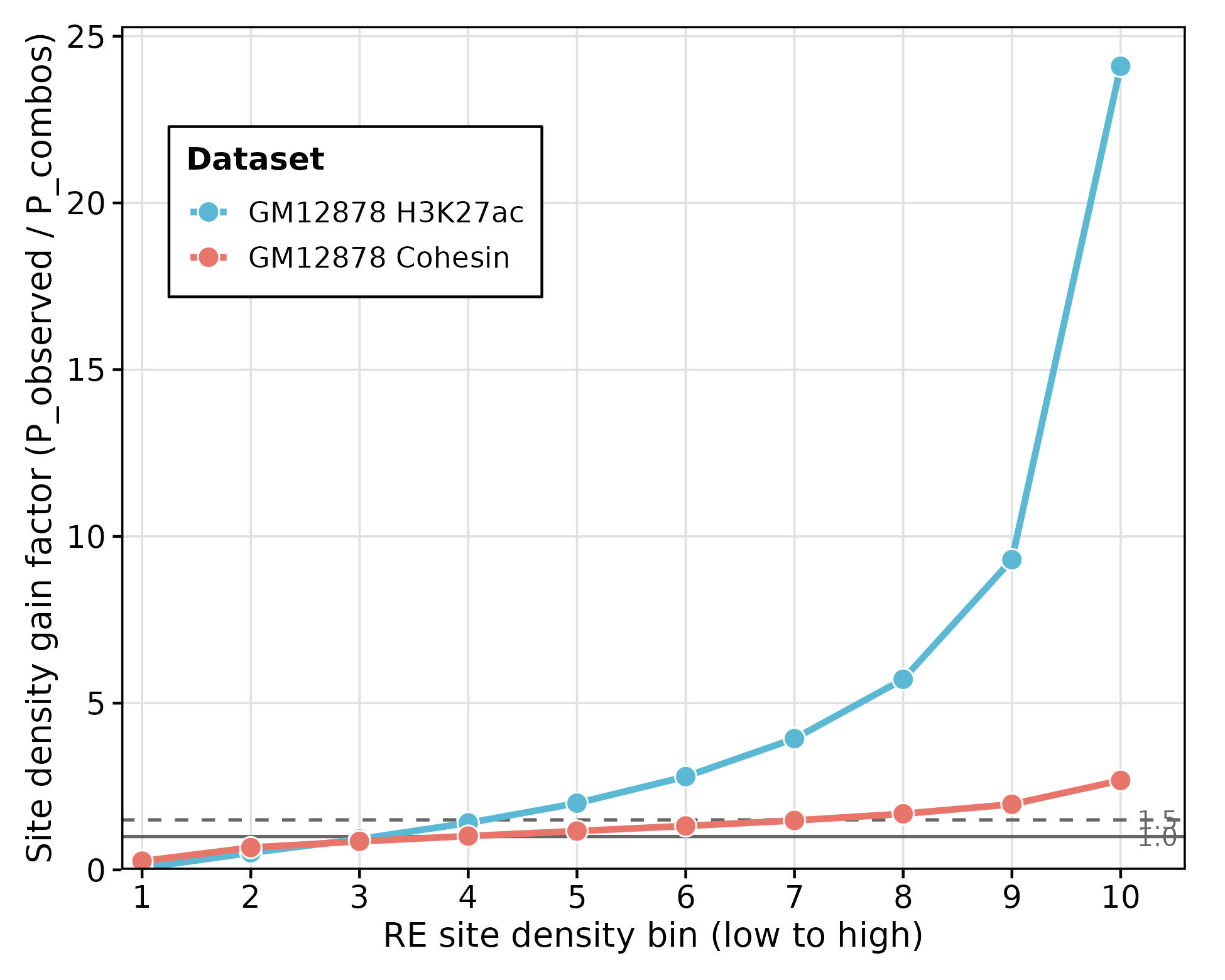


**Supplemental Figure S2.** Site density gain factors across restriction enzyme density deciles. Site density gain factor (ratio of observed to expected interaction probability) is plotted for GM12878 H3K27ac (blue) and GM12878 Cohesin (red) HiChIP. H3K27ac exhibits strong monotonic enrichment across deciles, representing a large dynamic range. Cohesin shows modest enrichment with a much smaller range. Horizontal dashed line indicates null expectation (site gain = 1). The difference in dynamic range between regulatory and structural contexts demonstrates differential behavior inconsistent with uniform technical bias.


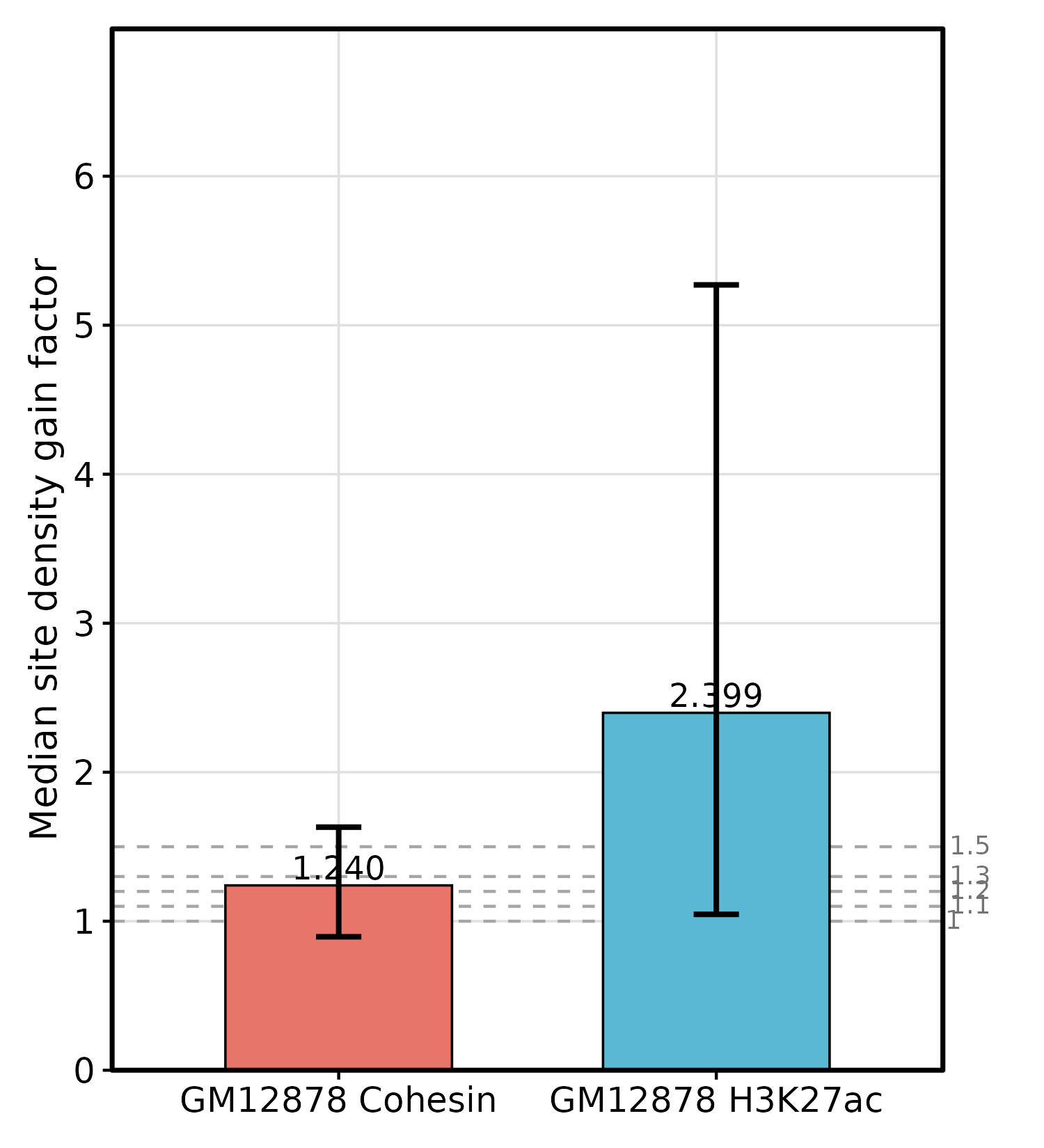


**Supplemental Figure S3.** Median site density gain factors with interquartile ranges. H3K27ac shows 2.40 times enrichment compared to 1.24 times for Cohesin (p < 2.2×10⁻¹⁶, Wilcoxon rank-sum test). Error bars represent 25th-75th percentiles across deciles. Horizontal reference lines mark integer fold-changes. The context-dependent enrichment patterns validate RE density as a biological signal reflecting chromatin accessibility rather than technical artifact.


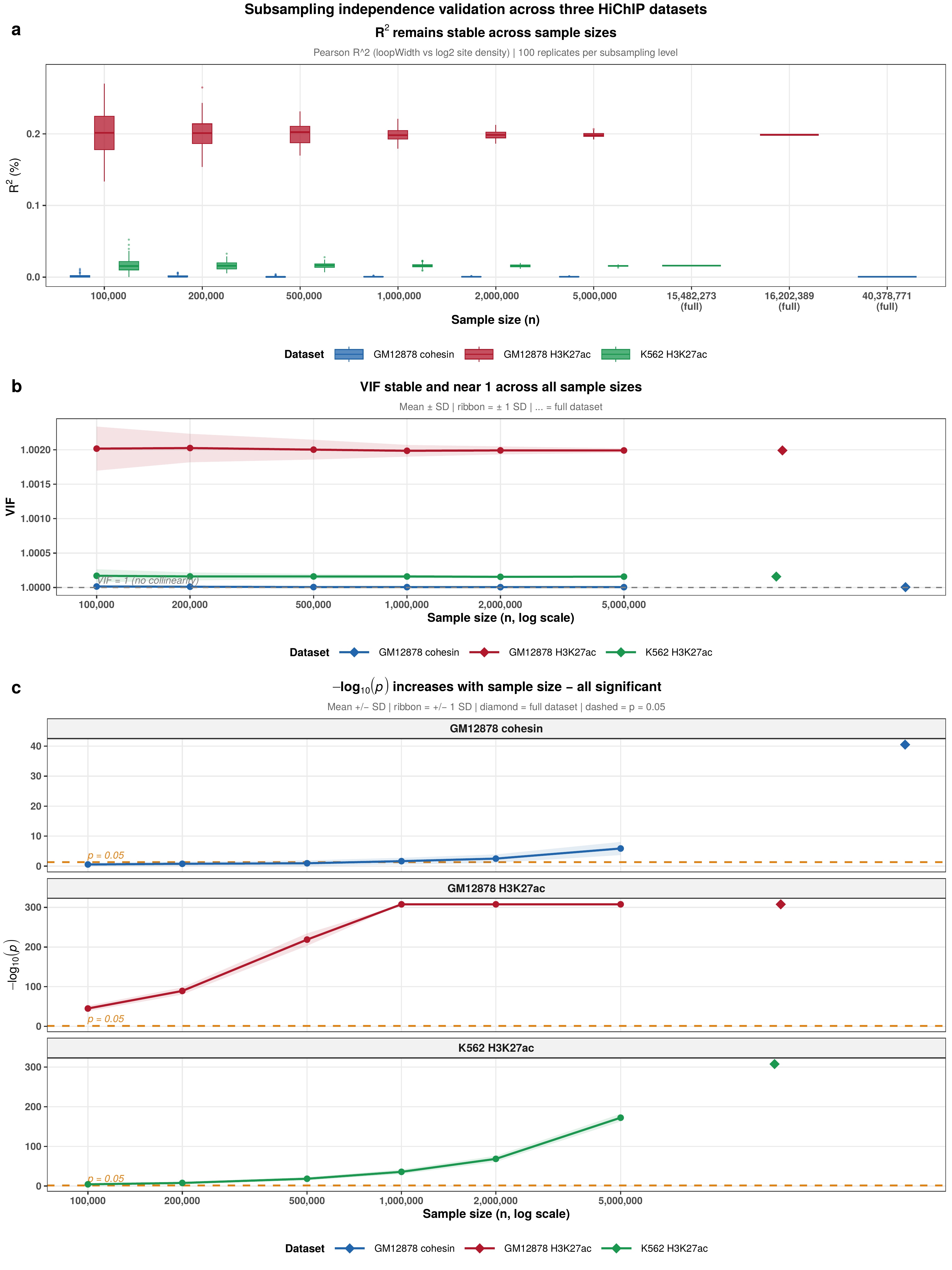


**Supplemental Figure S4.** Subsampling independence validation across three HiChIP datasets. Pearson R² (panel a), VIF (panel b), and −log10(p-value) (panel c) are shown as a function of sample size (n = 100,000 to full dataset, 100 replicates each) for GM12878 cohesin, GM12878 H3K27ac, and K562 H3K27ac. The key diagnostic is the R² trajectory: a true underlying correlation would produce stable or increasing R² as n grows, whereas a null effect produces near-zero stable R² while −log10(p) rises monotonically due to increased statistical power. Across all three datasets, R² remained ≤ 0.199% and VIF ≤ 1.002 at all sample sizes, confirming negligible collinearity. The significant p-value at full n (−log10(p) up to ~300) is a large-sample power artifact, not evidence of meaningful correlation.


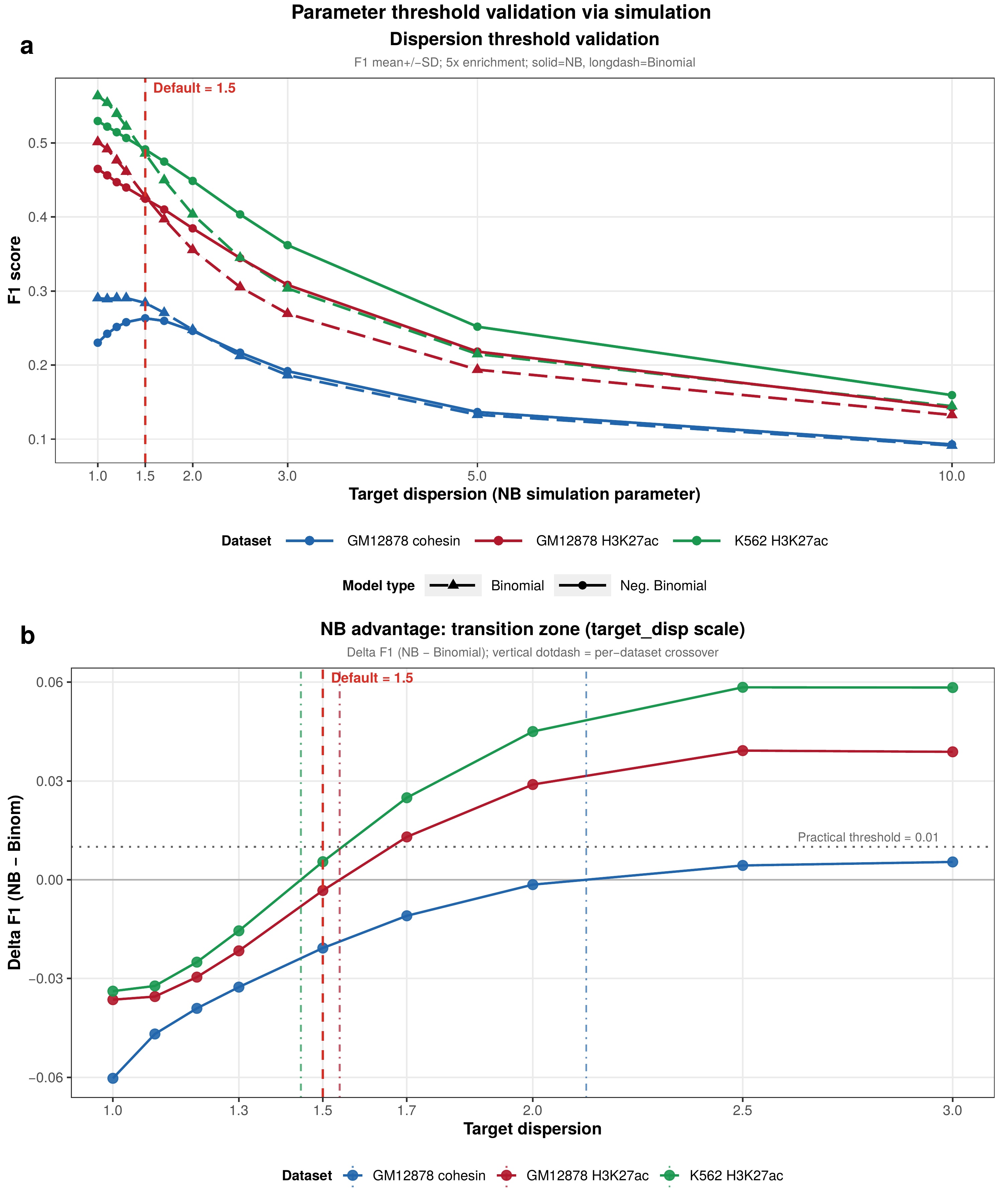


**Supplemental Figure S5.** Simulation-based threshold validation for adaptive distribution selection. (a) F1 scores for negative binomial (NB) and binomial models across target dispersions of 1.0–10.0 (5 times enrichment at true positives, 2% true positive fraction, 5 replicates per condition). NB begins to outperform binomial at dispersion ≈1.3–1.7 (dataset-dependent crossover). (b) ΔF1 (NB − binomial) as a function of target dispersion. ΔF1 exceeds 0.01 at dispersion > 1.5 across all three datasets, directly supporting the variance-to-mean threshold of 1.5 used in sintHiChIP.

**
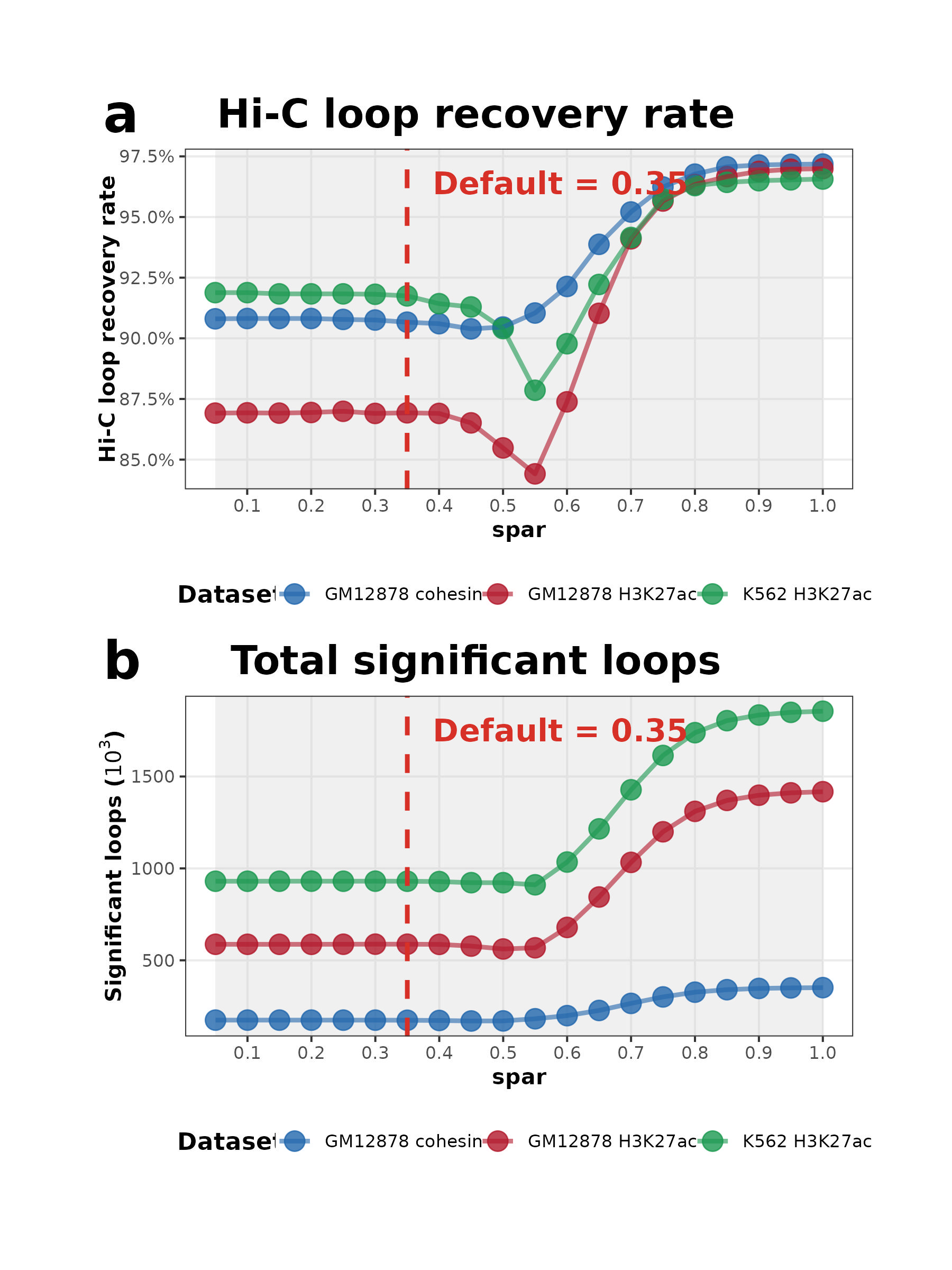
**

**Supplemental Figure S6.** Spar sensitivity analysis across 20 values (spar = 0.05–1.0) on all three HiChIP datasets. (a) Hi-C loop recovery rate as a function of spar. Recovery is stable within spar = 0.1–0.45 (<0.6 percentage points variation across all datasets) and degrades beyond spar = 0.5. (b) Significant loop counts as a function of spar. Counts remain stable within spar = 0.1–0.45 (<2.8% variation) and inflate sharply beyond spar = 0.5, reflecting over-smoothing of the background model.


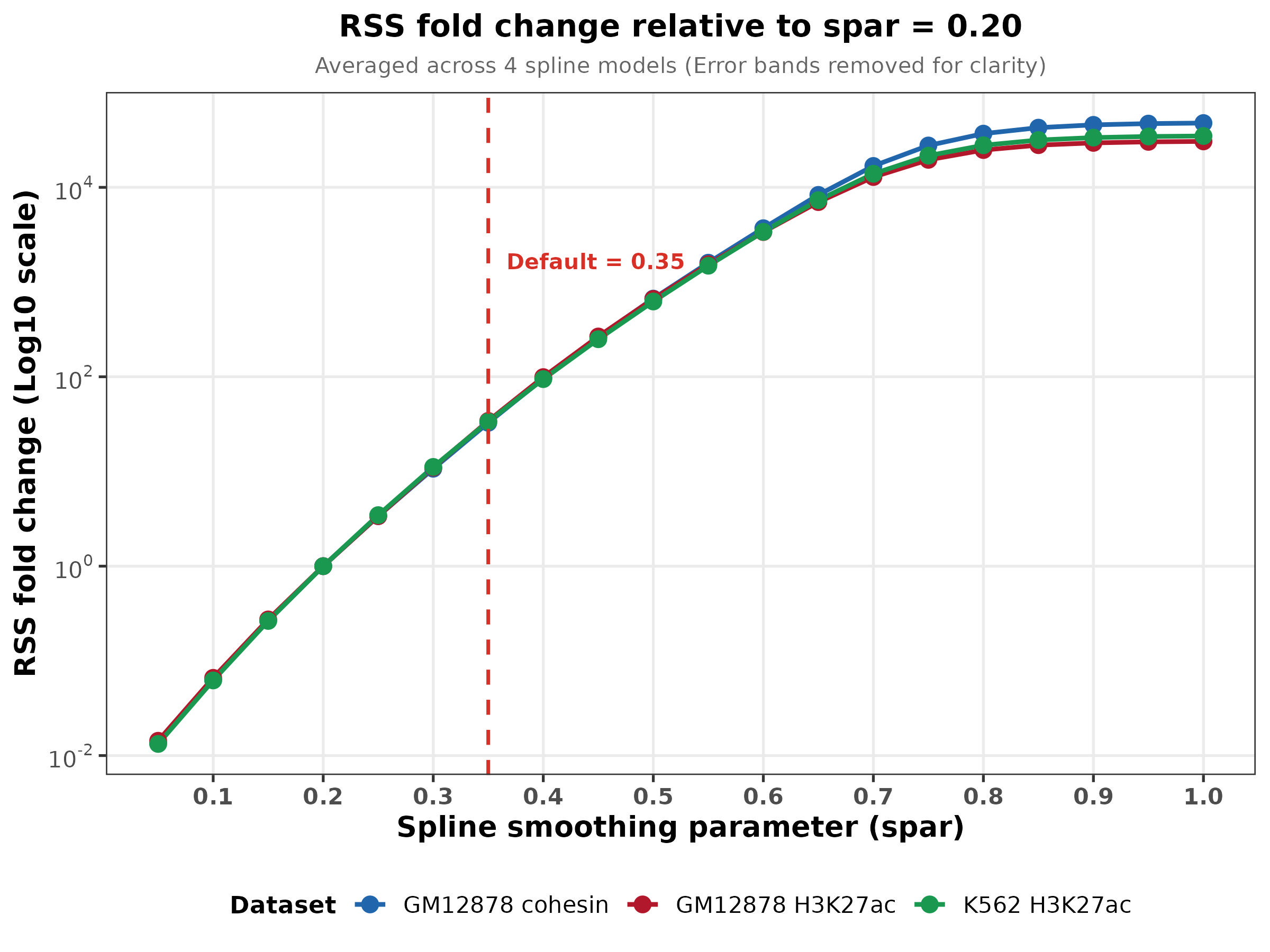


**Supplemental Figure S7.** Stability evaluation of the spline smoothing parameter spar. The y-axis displays the residual sum of squares (RSS) fold change on a log10 scale relative to a spar = 0.2 baseline, averaged across four spline models. The plot includes data from GM12878 cohesin, GM12878 H3K27ac, and K562 H3K27ac datasets. The default setting (spar = 0.35, vertical dashed line) maintains a moderate error rate. Conversely, assigning a spar value greater than 0.5 triggers an exponential increase in RSS. This severe deviation validates the chosen default parameter to prevent model underfitting.

**
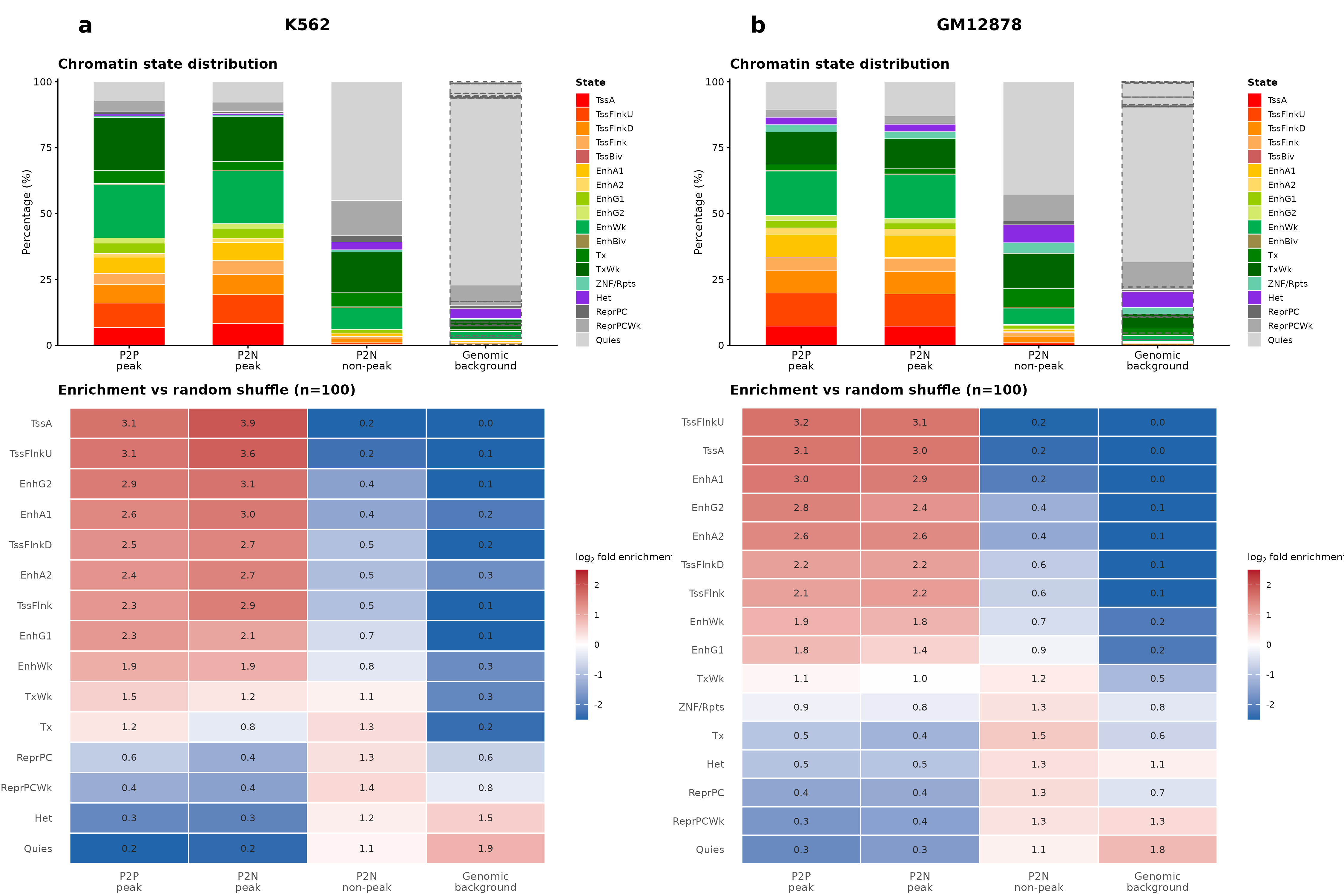
**

**Supplemental Figure S8.** ChromHMM chromatin state enrichment analysis of P2N non-peak anchors. Fold enrichment (observed/expected) for each of the 18 Roadmap Epigenomics chromatin states, computed against 100 size-matched random shuffles, shown for (a) K562 (E123) and (b) GM12878 (E116) cell lines. P2P peak and P2N peak anchors show strong enrichment for active states (TssA: 3.1–3.9 times; EnhA1: 2.6–3.0 times). P2N non-peak anchors show depletion of all active states (TssA: 0.17–0.20 times; EnhA1: 0.23–0.38 times) and modest enrichment for quiescent (Quies: 1.09–1.12 times), Polycomb-repressed (ReprPCWk: 1.26–1.37 times), and transcribed (Tx: 1.32–1.5 times) states. Cross-cell-type consistency demonstrates that these chromatin state associations are genuine biological features of P2N non-peak anchors. Error bars represent 95% confidence intervals from shuffles.

**
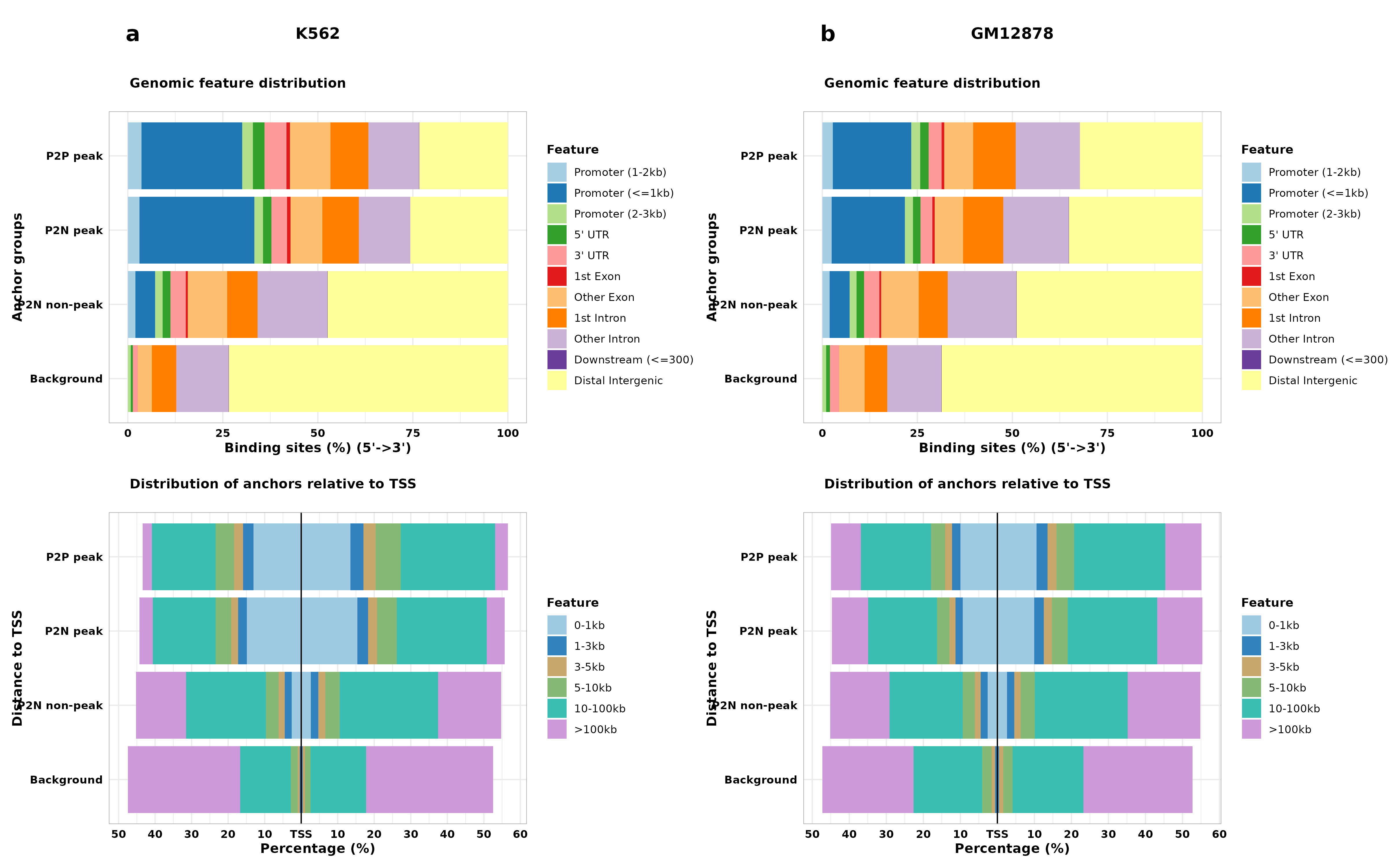
**

**Supplemental Figure S9.** Genomic feature annotation of interaction anchor groups in K562 and GM12878 cells.(a) K562 and (b) GM12878. Upper panels: Genomic feature distribution of four anchor groups (P2N peak regions, P2N non-peak regions, P2P regions, and genomic background), showing the percentage of anchors overlapping promoter regions (≤1 kb, 1–2 kb, 2–3 kb), UTRs, exons, introns, downstream regions (≤300 bp), and distal intergenic regions. Lower panels: Distribution of anchors relative to the nearest transcription start site (TSS), showing the percentage of anchors at distances of 0–1 kb, 1–3 kb, 3–5 kb, 5–10 kb, 10–100 kb, and >100 kb upstream and downstream of the TSS. The vertical black line indicates the TSS position.

**
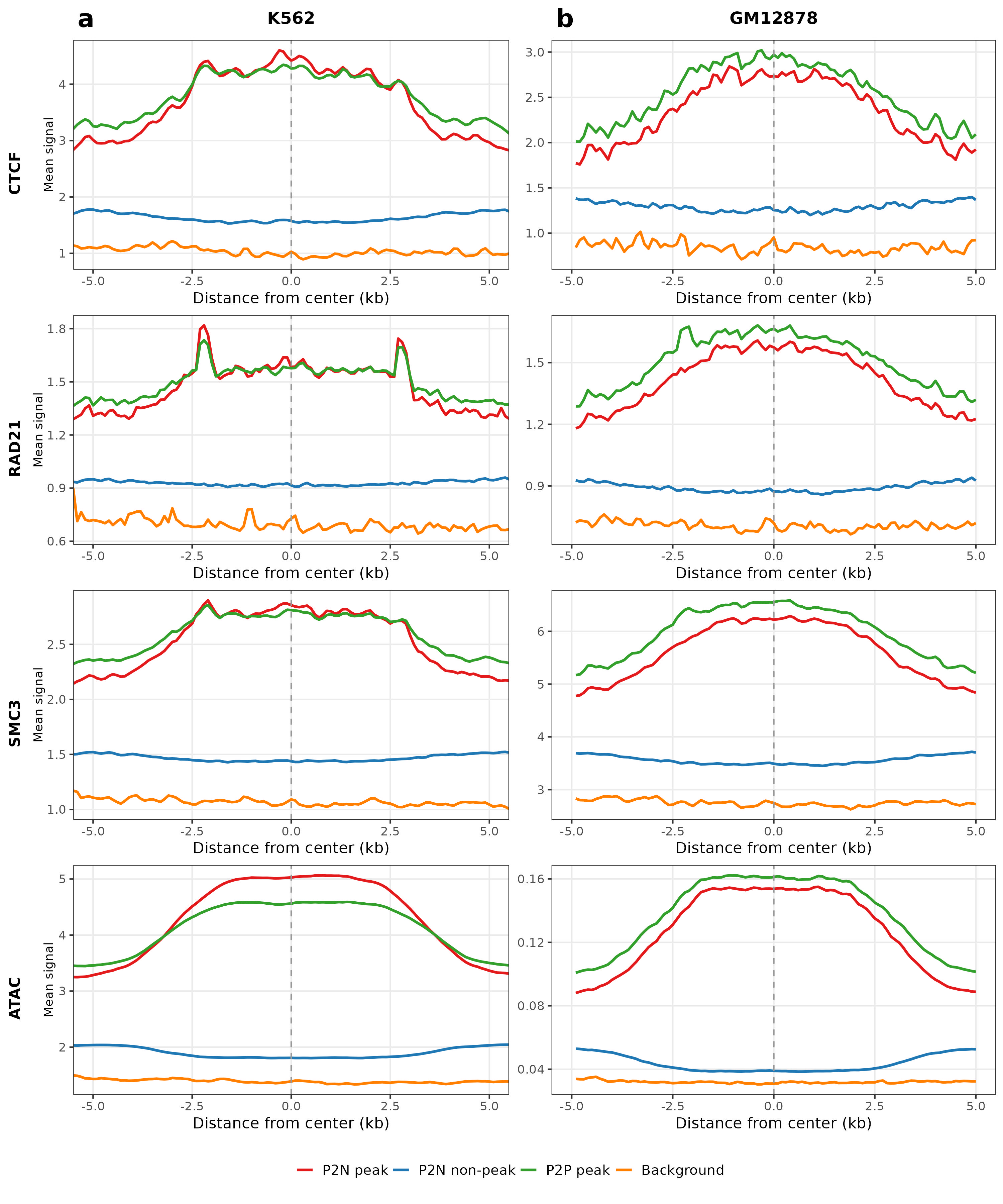
**

**Supplemental Figure S10.** Chromatin factor and accessibility enrichment profiles at interaction anchors. Average signal profiles of CTCF, RAD21, SMC3, and ATAC-seq centered on a ±5 kb window for four anchor groups, shown for (a) K562 and (b) GM12878. P2P and P2N peak anchors show the highest signal enrichment, while P2N non-peak anchors exhibit intermediate enrichment consistently and significantly above genomic background across all marks and both cell lines (all p < 0.001, Wilcoxon rank-sum tests), indicating sub-threshold but non-random biochemical occupancy at P2N non-peak anchors.


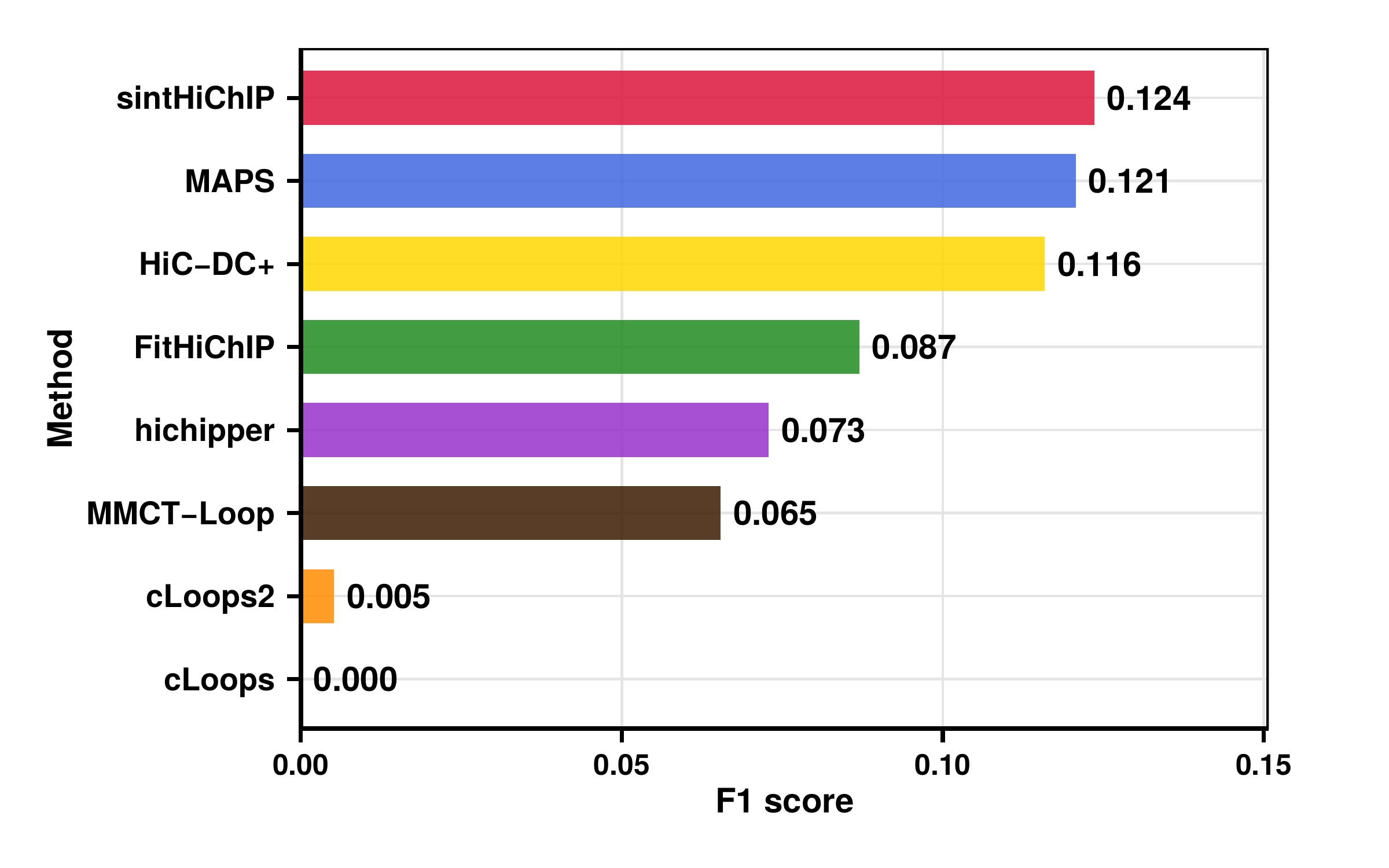


**Supplemental Figure S11.** F1 score comparison across methods. Horizontal bar plot quantifying precision-recall balance for K562 CRISPRi validation at q < 0.01. F1 scores range from 0.000 (cLoops) to 0.124 (sintHiChIP). sintHiChIP achieved the highest F1 score of 0.124, surpassing MAPS (0.121), HiC-DC+ (0.116), FitHiChIP (0.087), hichipper (0.073), MMCT-Loop (0.065), cLoops2 (0.005) and cLoops (0).

# References

1. Servant N, Varoquaux N, Lajoie BR, et al. 2015. HiC-Pro: an optimized and flexible pipeline for Hi-C data processing. Genome Biol 16: 259. doi:10.1186/s13059-015-0831-x

2. Bhattacharyya S, Chandra V, Vijayanand P, et al. 2019. Identification of significant chromatin contacts from HiChIP data by FitHiChIP. Nat Commun 10: 4221. doi:10.1038/s41467-019-11950-y

3. Zhang Y, Liu T, Meyer CA, et al. 2008. Model-based analysis of ChIP-Seq (MACS). Genome Biol 9: R137. doi:10.1186/gb-2008-9-9-r137

4. Juric I, Yu M, Abnousi A, et al. 2019. MAPS: model-based analysis of long-range chromatin interactions from PLAC-seq and HiChIP experiments. PLoS Comput Biol 15: e1006982. doi:10.1371/journal.pcbi.1006982

5. Lareau CA, Aryee MJ. 2018. hichipper: a preprocessing pipeline for calling DNA loops from HiChIP data. Nat Methods 15: 155-156. doi:10.1038/nmeth.4583

6. Sahin M, Wong W, Zhan Y, et al. 2021. HiC-DC+ enables systematic 3D interaction calls and differential analysis for Hi-C and HiChIP. Nat Commun 12: 3366. doi:10.1038/s41467-021-23749-x

7. Tang L, Liao J, Hill MC, et al. 2024. MMCT-Loop: a mix model-based pipeline for calling targeted 3D chromatin loops. Nucleic Acids Res 52: e25. doi:10.1093/nar/gkae029

8. Cao Y, Chen Z, Chen X, et al. 2020. Accurate loop calling for 3D genomic data with cLoops. Bioinformatics 36: 666-675. doi:10.1093/bioinformatics/btz664

9. Cao Y, Liu S, Ren G, et al. 2022. cLoops2: a full-stack comprehensive analytical tool for chromatin interactions. Nucleic Acids Res 50: 57-71. doi:10.1093/nar/gkab1155

10. Mumbach MR, Satpathy AT, Boyle EA, et al. 2017. Enhancer connectome in primary human cells identifies target genes of disease-associated DNA elements. Nat Genet 49: 1602-1612. doi:10.1038/ng.3963

11. Fulco CP, Nasser J, Jones TR, et al. 2019. Activity-by-contact model of enhancer-promoter regulation from thousands of CRISPR perturbations. Nat Genet 51: 1664-1669. doi:10.1038/s41588-019-0538-0

12. Roadmap Epigenomics Consortium, Kundaje A, Meuleman W, et al. 2015. Integrative analysis of 111 reference human epigenomes. Nature 518: 317-330. doi:10.1038/nature14248

13. Quinlan AR, Hall IM. 2010. BEDTools: a flexible suite of utilities for comparing genomic features. Bioinformatics 26: 841-842. doi:10.1093/bioinformatics/btq033

14. Yu G, Wang LG, He QY. 2015. ChIPseeker: an R/Bioconductor package for ChIP peak annotation, comparison and visualization. Bioinformatics 31: 2382-2383. doi:10.1093/bioinformatics/btv145

15. Ramírez F, Ryan DP, Grüning B, et al. 2016. deepTools2: a next generation web server for deep-sequencing data analysis. Nucleic Acids Res 44: W160–W165. doi:10.1093/nar/gkw257

16. Mumbach MR, Rubin AJ, Flynn RA, et al. 2016. HiChIP: efficient and sensitive analysis of protein-directed genome architecture. Nat Methods 13: 919-922. doi:10.1038/nmeth.3999

17. Rao SSP, Huntley MH, Durand NC, et al. 2014. A 3D map of the human genome at kilobase resolution reveals principles of chromatin looping. Cell 159: 1665-1680. doi:10.1016/j.cell.2014.11.021

18. GTEx Consortium. 2020. The GTEx Consortium atlas of genetic regulatory effects across human tissues. Science 369: 1318-1330. doi:10.1126/science.aaz1776

19. Heidari N, Phanstiel DH, He C, et al. 2014. Genome-wide map of regulatory interactions in the human genome. Genome Res 24: 1905-1917. doi:10.1101/gr.176586.114

20. ENCODE Project Consortium. 2012. An integrated encyclopedia of DNA elements in the human genome. Nature 489: 57-74. doi:10.1038/nature11247

21. Grau J, Grosse I, Keilwagen J. 2015. PRROC: computing and visualizing precision-recall and receiver operating characteristic curves in R. Bioinformatics 31: 2595-2597. doi:10.1093/bioinformatics/btv153
