# Supplementary material for: Accurate and efficient HiChIP interaction detection by modeling restriction enzyme cut site density as biological signal
Source: Brief Bioinform. 2026 Jun 8;27(3):bbag292. doi: 10.1093/bib/bbag292 (PMC13245736; doi:10.1093/bib/bbag292)
Supplement: Supplementary_materials_bbag292 [file supplementary_materials_bbag292.zip › Supplementary_materials_bbag292_legends.docx]

# Supplemental Tables

**Supplemental Table S1.** HiChIP sequencing data summary.

Summary of HiChIP datasets used in this study, including three cell types (GM12878 cohesin, GM12878 H3K27ac, K562 H3K27ac), individual biological replicates, and combined datasets. Total Valid Pairs represents the number of high-quality, long-range chromatin interaction pairs after duplicate removal and filtering (MAPSQUERRY-compliant). References: 1 Mumbach et al. 2016; 2 Mumbach et al. 2017.

**Supplemental Table S2.** Decomposition of sintHiChIP predictions into P2P and P2N interaction classes.

Recovery of gold-standard interactions by sintHiChIP partitioned into peak-to-peak (P2P) and peak-to-non-peak (P2N) categories. P2P interactions: both anchors overlap called ChIP peaks. P2N interactions: one anchor overlaps peak, the other anchor in non-peak region. Values represent percentages of total gold-standard loops, with absolute counts in parentheses. Gold-standard validation sets: Hi-C HiCCUPS loops (Rao et al. 2014) and ChIA-PET RAD21 loops (Bhattacharyya et al. 2019), filtered for 20 kb to 2 Mb distance range.

**Supplemental Table S3.** Aggregate peak analysis of predicted HiChIP loops validated against independent Hi-C data.

Aggregate peak analysis (APA) was performed on top-ranked loop sets using orthogonal Hi-C datasets at 5 kb and 10 kb resolution. P2LL (APA score) quantifies focal enrichment of Hi-C signals at predicted loop anchors relative to local background. R (enrichment ratio) measures overall interaction strength across anchor neighborhoods. Higher values indicate stronger validation of predicted loops.

**Supplemental Table S4.** CRISPRi-FlowFISH functional validation of predicted enhancer-promoter loops.

Performance metrics for identifying functional enhancer-promoter pairs validated by CRISPRi-FlowFISH experiments in K562 cells. Dataset contains 4,578 candidate pairs with 123 functional pairs showing significant CRISPRi effects (adjusted p < 0.05, |effect| > 0). Precision = proportion of functional pairs among detected pairs; Recall = proportion of detected functional pairs among all functional pairs; F1 = harmonic mean of precision and recall.

**Supplemental Table S5.** Cell-type-specific pathway enrichment of genes connected by eQTL regulatory loops.

Pathway enrichment analysis for genes connected by eQTL-associated regulatory loops in GM12878 cells, focusing on B cell and lymphoblastoid cell-specific pathways (q < 0.1). Analysis performed using Enrichr-KG. Pathways include BCR signaling components, B cell developmental phenotypes, cytokine responses, and immune activation signatures characteristic of lymphoblastoid cells. Representative genes show key pathway members identified by each method. Pathway Specificity Categories: Core represents GM12878/B cell specific pathways (BCR signaling, Fc receptor signaling, IgM level). Partial represents Partially related pathways (GTPase signaling, IL-6 response). None represents Non-specific pathways

**Supplemental Table S6.** eQTL regulatory loop metrics from GM12878 H3K27ac HiChIP analysis.

Summary statistics for eQTL-associated regulatory loops identified from GM12878 H3K27ac HiChIP data integrated with GTEx v10 eQTL data. Regulatory loops defined as interactions where eQTL variants (q < 0.05) map within ±10 kb of one anchor and target genes overlap the opposing anchor (20 kb to 1.5 Mb range, loop q < 0.01).

**Supplemental Table S7.** Runtime performance of HiChIP loop calling methods.

Wall-clock execution time measured from process initiation to completion for individual biological replicates and combined samples. All methods configured with identical parameters: 20 kb to 2 Mb distance range, FDR < 0.01. Benchmarking performed on AMD EPYC 7513 processor (32 cores, 64 threads), 258 GB RAM. HiC-DC+ tested with both single-core and 8-core configurations. Runtime values reported in minutes. MMCT-Loop, MAPS, cLoops, and cLoops2 both exceeded the 2-hour runtime threshold across all samples using 40 threads and are therefore reported as >120 min.

**Supplemental Table S8.** Peak memory consumption of HiChIP loop calling methods.

Peak memory usage monitored continuously via /proc/[pid]/status at 1-second sampling intervals throughout execution. All methods configured with identical parameters: 20 kb to 2 Mb distance range, FDR < 0.01. Benchmarking performed on AMD EPYC 7513 processor (32 cores, 64 threads), 258 GB RAM. Memory values reported in gigabytes (GB) using 1024³ byte conversion. For hichipper, individual replicate values represent dataset-level measurements as hichipper processes all samples within a dataset simultaneously. MMCT-Loop, MAPS, cLoops, and cLoops2 exceeded 40 GB memory limit across all samples using 40 threads and are therefore reported as >40 GB;

**Supplemental Table S9.** Processing stage time distribution for sintHiChIP.

Breakdown of execution time across three processing stages in global mode: Step 1 (matrix generation and peak filtering), Step 2 (statistical significance testing), Step 3 (browser track generation). Time values reported in seconds. Percentages show proportion of total runtime for each stage.

# Supplemental Figures

**Supplemental Figure S1.** Statistical framework validation and model characteristics.

sintHiChIP probability model components and statistical independence verification for GM12878 H3K27ac HiChIP genome-wide analysis. (a) Distribution of log2 joint cut site density across chromatin interactions. Histogram (blue bars) shows empirical frequency distribution; red curve represents fitted kernel density estimate. Distribution exhibits bell-shaped pattern centered around -7.5, spanning approximately 3 orders of magnitude. (b) Scatter plots quantify the relationship between genomic distance and joint site density; the shared variance is strictly bounded (R² = 0.00199, p < 2.2×10⁻¹⁶, VIF = 1.002) (c, d) Observed models capture the marginal effects of individual covariates on PET probability: interaction probability decays with genomic distance (c) and increases with cut site density (d). (e, f) Combo models characterize the joint background expectation across all possible anchor pair combinations; curve trajectories are inverted relative to the observed models. Across all four panels, empirical contact frequencies tightly trace the fitted splines.

**Supplemental Figure S2.** Site density gain factors across restriction enzyme density deciles.

Site density gain factor (ratio of observed to expected interaction probability) is plotted for GM12878 H3K27ac (blue) and GM12878 Cohesin (red) HiChIP. H3K27ac exhibits strong monotonic enrichment across deciles, representing a large dynamic range. Cohesin shows modest enrichment with a much smaller range. Horizontal dashed line indicates null expectation (site gain = 1). The difference in dynamic range between regulatory and structural contexts demonstrates differential behavior inconsistent with uniform technical bias.

**Supplemental Figure S3.** Median site density gain factors with interquartile ranges.

H3K27ac shows 2.40 times enrichment compared to 1.24 times for Cohesin (p < 2.2×10⁻¹⁶, Wilcoxon rank-sum test). Error bars represent 25th-75th percentiles across deciles. Horizontal reference lines mark integer fold-changes. The context-dependent enrichment patterns validate RE density as a biological signal reflecting chromatin accessibility rather than technical artifact.

**Supplemental Figure S4.** Subsampling independence validation across three HiChIP datasets.

Pearson R² (panel a), VIF (panel b), and −log10(p-value) (panel c) are shown as a function of sample size (n = 100,000 to full dataset, 100 replicates each) for GM12878 cohesin, GM12878 H3K27ac, and K562 H3K27ac. The key diagnostic is the R² trajectory: a true underlying correlation would produce stable or increasing R² as n grows, whereas a null effect produces near-zero stable R² while −log10(p) rises monotonically due to increased statistical power. Across all three datasets, R² remained ≤ 0.199% and VIF ≤ 1.002 at all sample sizes, confirming negligible collinearity. The significant p-value at full n (−log10(p) up to ~300) is a large-sample power artifact, not evidence of meaningful correlation.

**Supplemental Figure S5.** Simulation-based threshold validation for adaptive distribution selection.

(a) F1 scores for negative binomial (NB) and binomial models across target dispersions of 1.0–10.0 (5 times enrichment at true positives, 2% true positive fraction, 5 replicates per condition). NB begins to outperform binomial at dispersion ≈1.3–1.7 (dataset-dependent crossover). (b) ΔF1 (NB − binomial) as a function of target dispersion. ΔF1 exceeds 0.01 at dispersion > 1.5 across all three datasets, directly supporting the variance-to-mean threshold of 1.5 used in sintHiChIP.

**Supplemental Figure S6.** Spar sensitivity analysis across 20 values (spar = 0.05–1.0) on all three HiChIP datasets.

(a) Hi-C loop recovery rate as a function of spar. Recovery is stable within spar = 0.1–0.45 (<0.6 percentage points variation across all datasets) and degrades beyond spar = 0.5. (b) Significant loop counts as a function of spar. Counts remain stable within spar = 0.1–0.45 (<2.8% variation) and inflate sharply beyond spar = 0.5, reflecting over-smoothing of the background model.

**Supplemental Figure S7.** Stability evaluation of the spline smoothing parameter spar.

The y-axis displays the residual sum of squares (RSS) fold change on a log10 scale relative to a spar = 0.2 baseline, averaged across four spline models. The plot includes data from GM12878 cohesin, GM12878 H3K27ac, and K562 H3K27ac datasets. The default setting (spar = 0.35, vertical dashed line) maintains a moderate error rate. Conversely, assigning a spar value greater than 0.5 triggers an exponential increase in RSS. This severe deviation validates the chosen default parameter to prevent model underfitting.

**Supplemental Figure S8.** ChromHMM chromatin state enrichment analysis of P2N non-peak anchors.

Fold enrichment (observed/expected) for each of the 18 Roadmap Epigenomics chromatin states, computed against 100 size-matched random shuffles, shown for (a) K562 (E123) and (b) GM12878 (E116) cell lines. P2P peak and P2N peak anchors show strong enrichment for active states (TssA: 3.1–3.9 times; EnhA1: 2.6–3.0 times). P2N non-peak anchors show depletion of all active states (TssA: 0.17–0.20 times; EnhA1: 0.23–0.38 times) and modest enrichment for quiescent (Quies: 1.09–1.12 times), Polycomb-repressed (ReprPCWk: 1.26–1.37 times), and transcribed (Tx: 1.32–1.54 times) states. Cross-cell-type consistency demonstrates that these chromatin state associations are genuine biological features of P2N non-peak anchors. Error bars represent 95% confidence intervals from shuffles.

**Supplemental Figure S9.** Genomic feature annotation of interaction anchor groups in K562 and GM12878 cells.

(a) K562 and (b) GM12878. Upper panels: Genomic feature distribution of four anchor groups (P2N peak regions, P2N non-peak regions, P2P regions, and genomic background), showing the percentage of anchors overlapping promoter regions (≤1 kb, 1–2 kb, 2–3 kb), UTRs, exons, introns, downstream regions (≤300 bp), and distal intergenic regions. Lower panels: Distribution of anchors relative to the nearest transcription start site (TSS), showing the percentage of anchors at distances of 0–1 kb, 1–3 kb, 3–5 kb, 5–10 kb, 10–100 kb, and >100 kb upstream and downstream of the TSS. The vertical black line indicates the TSS position.

**Supplemental Figure S10.** Chromatin factor and accessibility enrichment profiles at interaction anchors.

Average signal profiles of CTCF, RAD21, SMC3, and ATAC-seq centered on a ±5 kb window for four anchor groups, shown for (a) K562 and (b) GM12878. P2P and P2N peak anchors show the highest signal enrichment, while P2N non-peak anchors exhibit intermediate enrichment consistently and significantly above genomic background across all marks and both cell lines (all p < 0.001, Wilcoxon rank-sum tests), indicating sub-threshold but non-random biochemical occupancy at P2N non-peak anchors.

**Supplemental Figure S11.** F1 score comparison across methods.

Horizontal bar plot quantifying precision-recall balance for K562 CRISPRi validation at q < 0.01. F1 scores range from 0.000 (cLoops) to 0.124 (sintHiChIP). sintHiChIP achieved the highest F1 score of 0.124, surpassing MAPS (0.121), HiC-DC+ (0.116), FitHiChIP (0.087), hichipper (0.073), MMCT-Loop (0.065), cLoops2 (0.005) and cLoops (0).
